# Global Research Progress on Radiofrequency Ablation in Cardiology

**DOI:** 10.1097/MS9.0000000000002858

**Published:** 2025-01-31

**Authors:** Chukwuka Elendu, Nkechi P. Ogwu, Alexander U. Okatta, Eunice K. Omeludike, Emmanuel C. Ogelle, Babajide T. Obidigbo, Mary C. Joseph, Emmanuella I Osamuyi, Afeez O. Ogidan, Klein A. Jingwa, Abdul-Rahaman A Ottun, Mariam M.F. Eldorghamy, Tuvakbibi Gurbanova, Fathy E.A.E. Soltan, Umesh Bhadana, Vaibhav S. Nasre, Cyrus P. Yadav, Rishabh Jaiswal

**Affiliations:** a Federal University Teaching Hospital, Owerri, Nigeria; b Odessa National Medical University, Odessa, Ukraine; c Imo State University, Owerri, Nigeria; d Piedmont Athens Regional Medical Centre, Athens, Georgia; e Blessed Specialist Hospital, Onitsha, Nigeria; f York and Scarborough Teaching Hospital NHS Foundation Trust, York, United Kingdom; g Ivan Horbachevsky Ternopil National Medical University, Ukraine; h Bingham University Teaching Hospital, Jos, Nigeria; i Olabisi Onabanjo University, Ago Iwoye, Nigeria; j Kazan state Medical University, Kazan, Russia

**Keywords:** cardiac arrhythmias, cardiology, clinical applications, global research, radiofrequency ablation

## Abstract

Radiofrequency ablation (RFA) has become a cornerstone in treating cardiac arrhythmias, offering a minimally invasive approach to managing conditions such as atrial fibrillation, ventricular tachycardia, and other rhythm disorders. The historical evolution of RFA, from its early inception to its current state, underscores the technological advancements that have significantly enhanced its efficacy and safety. Global trends indicate a steady increase in the adoption of RFA, with notable research contributions from North America, Europe, and Asia. Comparative studies reveal outcome variability driven by differences in patient populations, procedural techniques, and healthcare infrastructures. Despite its success, RFA faces challenges, including complications related to the procedure, patient selection, and long-term efficacy. Emerging technologies, such as integrating artificial intelligence and enhanced imaging modalities, hold promise for overcoming these barriers and further refining the procedure. Gaps in current research are identified, particularly in understanding the long-term outcomes of RFA and its application in complex arrhythmias. The critical role of RFA in modern cardiology is emphasized, along with the potential for future innovations that could expand its therapeutic utility. This synthesis of the latest evidence provides valuable insights for optimizing the use of RFA in managing cardiac arrhythmias.

## Public summary

Radiofrequency ablation (RFA) has become a game-changer in cardiology, offering hope and relief to millions suffering from heart rhythm disorders known as arrhythmias. Imagine a heart that occasionally skips a beat or races uncontrollably—these are the challenges faced by those with arrhythmias. RFA steps in as a precise, minimally invasive technique that uses heat generated by radio waves to target and destroy small areas of heart tissue, causing irregular rhythms and allowing the heart to beat normally again. Over the years, the field of RFA has witnessed incredible advancements. The evolution has been remarkable, from the early days of basic catheter designs to today’s sophisticated, high-tech tools that can map the heart in three dimensions. Doctors can now perform these procedures with greater accuracy and safety, significantly improving patient outcomes worldwide. But the journey doesn’t end here. Despite its success, RFA is not without challenges. Some patients still experience complications, and not everyone responds to treatment similarly. This has spurred researchers and clinicians to keep pushing the boundaries, exploring new technologies, and refining techniques to make RFA more effective and accessible. The future of RFA looks bright, with ongoing research focusing on personalized treatments, alternative energy sources like cryoablation, and innovative tools that promise to make procedures quicker, safer, and more effective.

## Introduction and background

RFA has been a transformative technique in managing cardiac arrhythmias, a prevalent condition associated with significant morbidity and mortality, since its introduction in the 1980s. Initially developed as an alternative to pharmacological treatments for drug-resistant arrhythmias, RFA offered a minimally invasive solution that disrupted abnormal electrical pathways within the heart by creating controlled lesions with high-frequency electrical energy^[1-3]^. Early RFA procedures were limited by technological constraints, including less sophisticated catheter designs and imaging capabilities, which impacted precision and safety. Over the past few decades, advances in catheter technology, imaging techniques, and procedural protocols have significantly enhanced the safety, efficacy, and applicability of RFA, solidifying its role as a primary treatment option for arrhythmias such as atrial fibrillation (AF) and ventricular tachycardia (VT). These advancements have not only broadened the range of arrhythmias treatable by RFA but have also reduced the risk of complications and improved long-term outcomes^[4-6]^. Despite these improvements, challenges remain, including arrhythmia recurrence, procedure-related complications, and the need for more data on long-term efficacy and cost-effectiveness, especially in complex cases. Emerging technologies, such as artificial intelligence (AI) and high-power, short-duration (HPSD) ablation techniques, hold promise for further enhancing precision and outcomes; however, these innovations are still under active investigation^[7-9]^. The primary aim of this literature review is to assess the global research progress on RFA in cardiology, focusing on its development, clinical applications, and emerging trends. This paper analyzes the evolution of RFA technology and techniques and their impact on managing cardiac arrhythmias, including AF, VT, and other rhythm disorders. By synthesizing the available evidence, the review identifies the key factors contributing to the success and limitations of RFA, such as procedural challenges, patient outcomes, and long-term efficacy. Additionally, the article explores the geographic variation in the adoption and outcomes of RFA, highlighting the contributions of different regions to advancing this technology. Finally, the review addresses current gaps in knowledge and proposes directions for future research to optimize the use of RFA in clinical practice and expand its therapeutic potential in cardiology.

## Information analyzing methods

**Figure 1.**
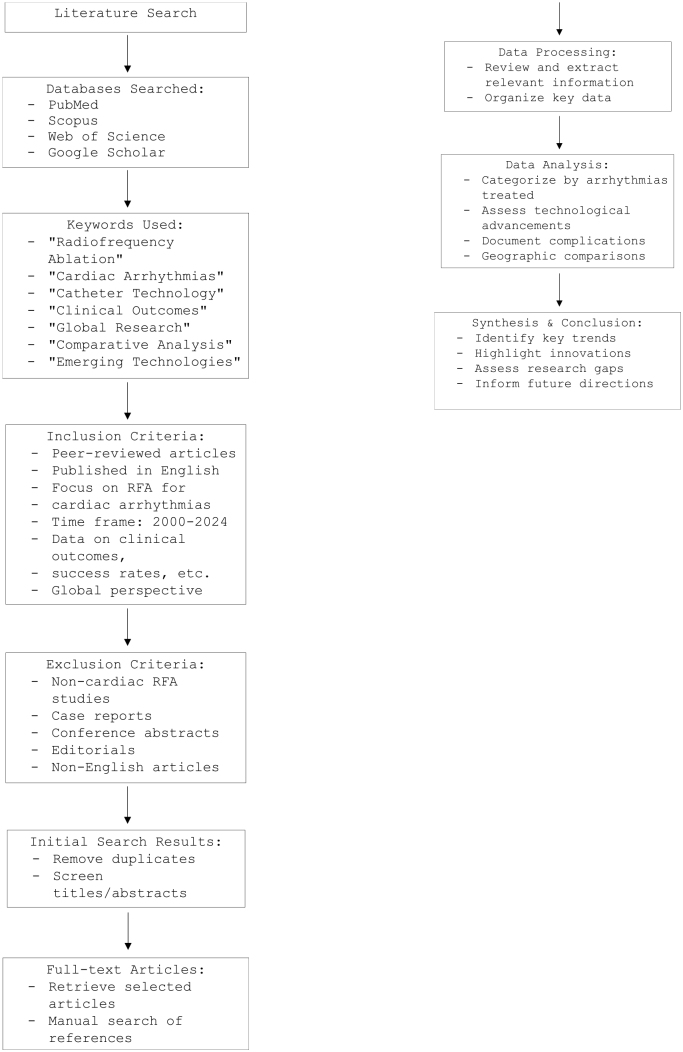
Literature Search Strategy for Review on RFA in Cardiology.

The data collection began with an extensive search across several scientific databases specifically chosen to ensure foundational and cutting-edge research coverage. As illustrated in Fig. 1, PubMed was prioritized for its comprehensive biomedical focus, capturing clinically relevant studies on RFA in cardiology. At the same time, Scopus and Web of Science were selected to broaden the scope of peer-reviewed literature across multiple disciplines. Google Scholar supplemented these with additional gray literature, capturing potentially relevant studies not indexed in other databases.

*The literature search strategy for this review on radiofrequency ablation (RFA) in cardiology involved comprehensive searches in PubMed, Scopus, Web of Science, and Google Scholar using keywords such as “Radiofrequency Ablation,” “Cardiac Arrhythmias,” “Catheter Technology,” “Clinical Outcomes,” “Global Research,” “Comparative Analysis,” and “Emerging Technologies.” Articles were included if they were peer-reviewed, published in English, focused on RFA for cardiac arrhythmias, and provided data on clinical outcomes or success rates from 2000 to 2024, with a global perspective. Studies on non-cardiac RFA, case reports, conference abstracts, editorials, and non-English articles were excluded. Initial search results were refined by removing duplicates and screening titles and abstracts. Selected articles were retrieved in full, and references were manually searched. Data extraction focused on relevant information, categorizing findings by arrhythmias treated, technological advancements, documented complications, and geographic comparisons. Finally, a synthesis identified key trends, innovations, and research gaps, providing insights for future research directions. [Authors’ Creations].*

To refine the search, specific keywords, and MeSH terms were selected for their ability to encompass established and emerging areas in RFA. Terms like “radiofrequency ablation” and “cardiac arrhythmias” targeted the core procedural focus. At the same time, “catheter technology,” “clinical outcomes,” and “comparative analysis” addressed specific clinical and comparative aspects central to understanding procedural success and technological variations. “Emerging technologies” and “global research” keywords captured recent innovations and global perspectives essential for a comprehensive review.

The time frame from 2000 to 2024 was chosen to include the most relevant and transformative period in RFA advancements, as key innovations in catheter design, energy delivery, and mapping systems emerged in the early 2000s. Only peer-reviewed articles published in English were considered to ensure high data quality and uniform interpretability. Inclusion criteria required studies on RFA for cardiac arrhythmias, presenting data on clinical outcomes, success rates, complications, or technological advancements. A global perspective or comparative analysis was also required to capture variations in RFA practices and innovations across different countries or regions.

Exclusion criteria were applied to filter out non-cardiac RFA studies, case reports, conference abstracts, editorials with limited data, and non-English publications, as these sources often lack the detailed data necessary for evaluating clinical outcomes and procedural advancements.

**Figure 2.**
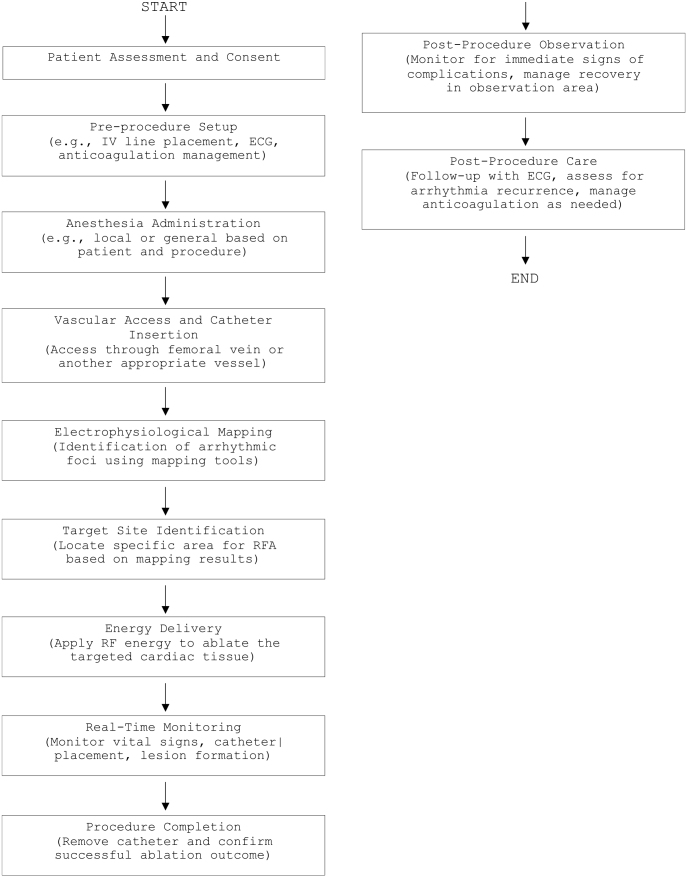
Procedural Workflow of RFA.

After the initial search, duplicate articles were removed, and titles and abstracts of remaining studies were screened against the inclusion criteria. Full-text articles were retrieved for studies meeting these criteria, and manual searches of selected articles’ reference lists helped identify additional relevant studies. A flowchart of the RFA process is included in Fig. 2 to outline the procedural workflow further, illustrating key steps from patient assessment to post-procedural care.

*This flowchart outlines a standard RFA process from patient assessment to post-procedural care. Each step is essential to ensure patient safety, procedural success, and effective recovery. Let me know if you need further details in any step. [Authors’ Creations].*

## Development of RFA technology

RFA has emerged as a pivotal technology in managing cardiac arrhythmias, revolutionizing the treatment landscape with its minimally invasive approach and efficacy in targeting abnormal electrical pathways within the heart. The development of RFA technology is rooted in a long history of scientific discovery and technological innovation, beginning with the foundational principles of radiofrequency energy application and evolving through successive waves of advancements in catheter design, imaging, and procedural techniques. Using radiofrequency energy to create controlled tissue destruction dates back to the early 20th century when it was first explored in neurosurgery. Harvey Cushing and his colleagues were among the pioneers who recognized the potential of high-frequency alternating current to create precise lesions in nervous tissue, a principle that laid the groundwork for future applications in other medical fields^[1,2]^. The extension of this concept to cardiology occurred several decades later, driven by the need for more effective treatments for arrhythmias that were refractory to pharmacological therapy and surgical interventions. The introduction of RFA into clinical cardiology can be traced to the late 1970s and early 1980s, a period marked by significant advancements in electrophysiology and the increasing use of catheter-based techniques for arrhythmia management. Direct current (DC) ablation was initially employed, which involved delivering a high-energy shock to ablate cardiac tissue. While effective in disrupting arrhythmic pathways, DC ablation was associated with a high incidence of complications, including barotrauma and myocardial perforation, due to the intense and uncontrolled nature of the energy delivery^[3,4]^. The limitations of DC ablation spurred the search for alternative energy sources that could provide more controlled and precise tissue ablation. Radiofrequency energy emerged as a promising alternative due to its ability to generate localized heat through the resistive heating of tissue, resulting in controlled coagulation necrosis. The use of radiofrequency energy for cardiac ablation was first described in animal models, where it demonstrated the ability to create discrete lesions with minimal collateral damage, a significant advantage over DC ablation^[5,6]^. These early studies provided the foundation for the first human applications of RFA, which were performed in the late 1980s by physicians such as Melvin Scheinman and others, who successfully used RFA to treat patients with refractory supraventricular tachycardia (SVT)^[7,8]^. The success of these initial procedures rapidly accelerated the development of RFA technology, leading to the introduction of dedicated radiofrequency generators and catheters designed specifically for cardiac ablation. The first-generation RFA catheters were relatively simple, consisting of a unipolar electrode connected to an external RF generator at the distal tip. The catheter was guided to the target site within the heart using fluoroscopic imaging, and radiofrequency energy was delivered to create the ablation lesion. While these early systems were effective, they had several limitations, including the inability to precisely control lesion size and depth and the challenges associated with visualizing the catheter position and assessing lesion formation in real time^[9,10]^. To address these challenges, subsequent developments in RFA technology focused on improving catheter design, energy delivery, and procedural guidance. One of the key innovations in this regard was the introduction of temperature-controlled ablation, which allowed for more precise regulation of the energy delivered to the tissue. By integrating thermocouples into the catheter tip, clinicians could monitor tissue temperature in real time and adjust the power output to maintain a consistent ablation temperature, thereby reducing the risk of overheating and collateral damage^[11,12]^. This advancement significantly improved the safety and efficacy of RFA, particularly in treating arrhythmias located in sensitive areas of the heart, such as near the AV node or within the pulmonary veins. In parallel with improvements in energy delivery, there were significant advancements in catheter technology. The development of steerable catheters with flexible, deflectable tips enhanced the ability of operators to navigate the complex anatomy of the heart and position the ablation electrode precisely at the target site. Additionally, the introduction of irrigated-tip catheters, which incorporate a saline irrigation system to cool the catheter tip during ablation, further enhanced lesion formation by preventing overheating and allowing for the delivery of higher energy levels without increasing the risk of tissue charring or coagulum formation^[13,14]^. These innovations in catheter design have been instrumental in expanding the scope of RFA to more complex arrhythmias, such as AF and VT, where creating larger and deeper lesions is often necessary. The evolution of imaging and mapping technologies has also played a crucial role in developing RFA. Initially, RFA procedures were guided primarily by fluoroscopy, which provided limited information on the heart’s three-dimensional (3D) structure and the precise location of the ablation catheter. The introduction of electroanatomic mapping systems, such as CARTO and EnSite, represented a significant breakthrough, enabling the creation of detailed 3D maps of the heart’s electrical activity. These systems use catheter-based sensors to track the position of the ablation catheter in real time, allowing operators to visualize the electrical signals and anatomical structures simultaneously. This integration of mapping and ablation has not only improved the accuracy and safety of RFA but has also facilitated the treatment of complex arrhythmias, such as those associated with scar tissue or multiple reentrant circuits^[15-17]^. Further advancements in imaging, such as intracardiac echocardiography (ICE) and magnetic resonance imaging (MRI), have enhanced the ability to visualize cardiac structures and assess lesion formation during and after ablation. ICE, in particular, has become a valuable tool in guiding RFA, providing real-time images of the catheter tip and surrounding tissue, and allowing for the assessment of complications such as pericardial effusion or thrombus formation^[18,19]^. MRI, while still primarily used for pre-and post-procedural assessment, holds the potential for real-time guidance of RFA in the future, offering the possibility of non-invasive lesion visualization and assessment of myocardial scarring^[20,21]^. The ongoing development of RFA technology has also been driven by a deeper understanding of the pathophysiology of arrhythmias and the identification of more precise ablation targets. For example, research into the mechanisms underlying AF has led to the widespread adoption of pulmonary vein isolation (PVI) as the cornerstone of AF ablation. The realization that AF is often triggered by ectopic foci within the pulmonary veins has shifted the focus of ablation from the atrial myocardium to the veins themselves, resulting in more effective and durable outcomes^[22,23]^. Similarly, recognizing the role of scar tissue in the genesis of ventricular arrhythmias has led to the development of substrate-based ablation strategies, where the ablation is targeted at the scar tissue and the surrounding area to eliminate the arrhythmogenic substrate^[24,25]^. In recent years, there has been growing interest in using HPSD ablation as an alternative to traditional low-power, long-duration ablation. The rationale behind HPSD is to create more efficient and homogeneous lesions by delivering higher energy levels over a shorter period. Preliminary studies have suggested that HPSD may offer several advantages, including shorter procedure times, reduced risk of complications, and improved lesion durability. However, further research is needed to establish the long-term outcomes of this approach^[26-28]^. Integrating AI and machine learning (ML) into RFA technology represents a promising frontier in the field. AI and ML have the potential to enhance various aspects of RFA, from pre-procedural planning to real-time decision-making during ablation. For instance, AI algorithms can analyze large datasets of patient information and procedural outcomes to identify predictors of success and guide personalized treatment strategies. Additionally, AI-driven mapping systems can assist operators in identifying critical areas for ablation and optimizing lesion placement, potentially reducing the risk of arrhythmia recurrence and improving long-term outcomes^[29-31]^. The development of AI-enhanced RFA is still in its early stages, but its potential to transform the field of electrophysiology is significant. Despite the substantial progress in RFA technology, several challenges and limitations remain. One of the key challenges is the variability in RFA outcomes, which can be influenced by a range of factors, including patient characteristics, the underlying arrhythmia substrate, and operator experience. For example, while RFA has shown high success rates in paroxysmal AF, the outcomes in persistent AF are less consistent, with higher rates of recurrence and the need for repeat procedures^[32,33]^. Similarly, the success of RFA in VT is often limited by the complexity of the arrhythmogenic substrate, particularly in patients with extensive myocardial scarring or non-ischemic cardiomyopathy^[9,34]^. Ongoing research is focused on addressing these challenges through the development of more sophisticated mapping and imaging techniques, as well as the refinement of ablation strategies. Another area of active research is the exploration of alternative energy sources for ablation. While radiofrequency remains the most widely used energy source for cardiac ablation, other modalities, such as cryoablation, laser ablation, and ultrasound ablation, have been investigated as potential alternatives. Cryoablation, for instance, uses extreme cold to create lesions and has been shown to have a lower risk of complications such as pulmonary vein stenosis when used for PVI^[35,36]^. However, the larger size and slower formation of cryolesions pose certain limitations, particularly when precise lesion control is necessary. Despite these challenges, cryoablation has gained acceptance as an effective alternative in specific clinical scenarios, such as in the treatment of atrioventricular nodal reentrant tachycardia (AVNRT) and patients with high risk for thromboembolic complications during RFA^[37,38]^. Meanwhile, other energy sources like laser and ultrasound are still largely experimental, with ongoing studies exploring their efficacy, safety, and potential applications in cardiac ablation^[39,40]^. In addition to exploring alternative energy sources, there has been significant interest in developing hybrid ablation techniques, which combine different energy modalities or integrate surgical and catheter-based approaches. One such technique is the hybrid convergent procedure for AF, which combines minimally invasive epicardial ablation performed by a cardiac surgeon with endocardial ablation performed by an electrophysiologist. This approach aims to achieve more comprehensive lesion sets and better outcomes, particularly in patients with persistent or longstanding persistent AF^[41,42]^. Initial results from hybrid ablation procedures have been promising, showing higher success rates and lower arrhythmia recurrence than catheter ablation alone, although long-term data are still needed^[43,44]^. The field of RFA continues to evolve with the development of novel catheter designs and technologies to improve precision and efficacy. One notable innovation is contact force-sensing catheters, which provide real-time feedback on the force applied by the catheter tip against the cardiac tissue. Studies have demonstrated that optimizing contact force during RFA can improve lesion formation and reduce the risk of complications such as perforation or incomplete ablation^[45,46]^. This technology has been particularly beneficial in the ablation of AF, where consistent and adequate tissue contact is crucial for the success of PVI^[47,48]^. The advent of robotic-assisted systems for catheter ablation represents another significant milestone in developing RFA technology. Robotic systems offer enhanced precision and stability during ablation, allowing for more consistent lesion creation and reducing operator fatigue. These systems also facilitate the performance of complex ablation procedures that may be challenging with manual catheter manipulation. Early studies on robotic-assisted RFA have shown promising results, with comparable or superior outcomes to conventional catheter ablation. However, widespread adoption has been limited by the high cost and complexity of these systems^[49-51]^. Integrating data analytics and ML into the electrophysiology workflow will likely drive the next wave of innovation in RFA. By leveraging large datasets from previous procedures, ML algorithms can identify patterns and predictors of success or complications, aiding in pre-procedural planning and real-time decision-making. For instance, ML models have been developed to predict the likelihood of arrhythmia recurrence following ablation, enabling personalized risk stratification and tailored treatment approaches^[6,52,53]^. Moreover, the combination of ML with advanced imaging and mapping technologies has the potential to further enhance the precision and outcomes of RFA procedures. The growing body of evidence supporting the efficacy and safety of RFA has led to its widespread adoption in clinical practice, with a broadening of indications and patient populations. Initially used primarily for the treatment of SVTs, RFA is now a standard of care for a wide range of arrhythmias, including AF, VT, and accessory pathway-mediated arrhythmias^[54-56]^. This expansion has been driven by continuous improvements in technology and technique and by the increasing recognition of the limitations of pharmacological therapy in managing these conditions. The success of RFA in achieving long-term arrhythmia control with a relatively low risk of complications has made it an attractive option for both patients and clinicians. However, as RFA technology advances, areas still require further investigation and development. One such area is the durability of ablation lesions, particularly in AF. Despite initial success, many patients experience arrhythmia recurrence following AF ablation, necessitating repeat procedures^[57,58]^. This has prompted ongoing research into optimizing lesion formation, improving procedural techniques, and identifying better markers of successful ablation. Developing biomarkers and imaging modalities that can accurately predict long-term outcomes remains a critical area of focus, potentially improving patient selection and reducing the need for repeat ablations^[59,60]^. Another important consideration in the development of RFA technology is the management of complications. While RFA is generally safe, it is not without risks. Potential complications include cardiac perforation, thromboembolism, pulmonary vein stenosis, and esophageal injury, among others^[61-63]^. Advances in imaging and catheter technology have helped mitigate some of these risks, but ongoing vigilance and innovation are necessary to enhance the safety profile of RFA further. For example, esophageal temperature monitoring during AF ablation has been shown to reduce the incidence of esophageal injury. At the same time, techniques such as high-resolution imaging and real-time lesion assessment may help prevent other complications^[64,65]^. The role of RFA in treating ventricular arrhythmias, particularly in the context of structural heart disease, is another area of active research. While RFA has proven highly effective in the treatment of idiopathic VT, its success in patients with ischemic or non-ischemic cardiomyopathy is more variable^[66-68]^. This variability is often due to the complex and heterogeneous nature of the arrhythmogenic substrate in these patients, which may involve multiple reentrant circuits, areas of fibrosis, and scar-related arrhythmias. Efforts to improve outcomes in these challenging cases have focused on refining mapping and ablation techniques, such as the use of substrate modification and the targeting of late potentials, as well as the integration of imaging modalities like MRI to guide ablation^[9,30,69]^. The future of RFA technology is likely to be shaped by a combination of incremental improvements in existing technologies and the introduction of disruptive innovations. For instance, the development of novel energy sources, such as pulsed-field ablation (PFA), which uses high-voltage electric fields to create non-thermal lesions, has shown promise in preclinical studies and early clinical trials^[35,70]^. PFA offers several advantages over traditional RFA, including faster lesion creation, reduced risk of collateral damage, and the ability to selectively target myocardial tissue while sparing surrounding structures such as blood vessels and nerves^[71,72]^. If these benefits are confirmed in larger studies, PFA could represent a significant leap forward in cardiac ablation.

## Mechanisms and techniques

The fundamental mechanism of RFA involves using high-frequency alternating current to generate heat through resistive heating of the surrounding tissues. When a catheter tip delivers radiofrequency energy to the myocardium, it causes ion agitation within the tissue, which leads to frictional heating^[10]^. The heat generated results in thermal coagulation and necrosis of the targeted myocardial tissue, creating a lesion that disrupts the abnormal electrical pathways responsible for the arrhythmia. RFA aims to precisely create lesions in specific areas of the heart to prevent the propagation of arrhythmic signals without damaging surrounding structures. One critical factor determining the effectiveness of RFA is the temperature achieved at the catheter-tissue interface^[11]^. Typically, the temperature is controlled to remain between 50°C and 70°C. At temperatures below 50°C, the energy delivered may not be sufficient to cause permanent tissue damage, leading to the possibility of arrhythmia recurrence. Conversely, temperatures above 70°C can result in excessive tissue damage and an increased risk of complications such as steam pops, which occur when water within the tissue rapidly vaporizes, leading to an explosive release of energy that can cause tissue perforation or embolism. The depth and size of the lesions created by RFA are also influenced by several factors, including the power delivered, the duration of energy application, the contact force between the catheter and tissue, and the properties of the tissue itself. Higher power settings and longer application times can increase lesion size and depth, but they also carry a higher risk of complications^[12-14]^. Therefore, careful titration of energy delivery is crucial to optimize lesion formation while minimizing the risk of adverse events. One of the key challenges in RFA is ensuring adequate tissue contact throughout the procedure. Poor contact can result in inadequate lesion formation, which may lead to incomplete ablation and arrhythmia recurrence. To address this, modern RFA catheters are equipped with contact force sensors that provide real-time feedback on the force applied by the catheter tip against the myocardial tissue. Studies have shown that maintaining an optimal contact force (typically between 10 and 40 g) can significantly improve the success rate of RFA by ensuring more consistent and effective lesion formation^[15]^. Another technique developed to enhance the effectiveness of RFA is irrigated-tip catheter ablation. This technique continuously infuses saline through the catheter tip during energy delivery. The saline cools the catheter tip and the adjacent tissue, allowing higher power settings without overheating. This cooling effect helps prevent char formation on the catheter tip, which can act as an insulator and reduce energy delivery to the tissue. Irrigated-tip catheters have been shown to create deeper and wider lesions than non-irrigated catheters, making them particularly useful for ablating thicker myocardial tissue, such as that found in the ventricular myocardium^[16-18]^. The success of RFA also depends on the accurate identification and targeting of the arrhythmogenic substrate. Electroanatomical mapping systems have played a crucial role in improving the precision of RFA by allowing for detailed visualization of the cardiac anatomy and the electrical activity within the heart. These systems use data from the catheter to create a three-dimensional heart map, highlighting areas of abnormal electrical activity that can be targeted for ablation. The integration of advanced mapping techniques, such as voltage mapping and activation mapping, has further enhanced the ability of electrophysiologists to identify the critical sites responsible for arrhythmias. Voltage mapping is particularly useful in identifying regions of scar tissue or areas of low voltage, which are often the sources of reentrant circuits that sustain arrhythmias. By targeting these low-voltage areas, RFA can effectively disrupt the reentrant pathways and prevent the recurrence of arrhythmias^[19-21]^. Activation mapping, on the other hand, is used to track the spread of electrical impulses through the myocardium, helping to pinpoint the origin of focal arrhythmias such as premature ventricular contractions (PVCs) or atrial tachycardias. In addition to standard RFA techniques, several specialized ablation strategies have been developed for specific arrhythmias. For example, PVI is widely used for treating AF. AF is often triggered by ectopic foci within the pulmonary veins, and PVI involves the creation of circumferential lesions around the pulmonary vein ostia to isolate these foci from the rest of the atrium electrically^[22-24]^. The success of PVI depends on achieving complete electrical isolation of the pulmonary veins, which requires careful mapping and ablation to eliminate all potential conduction pathways. Another specialized technique is substrate modification, often used in treating VT in patients with structural heart disease. VT in these patients is typically sustained by reentrant circuits that form within areas of scar tissue. Substrate modification involves the strategic ablation of these areas to disrupt the reentrant circuits and prevent the initiation and maintenance of VT. This technique often requires a combination of voltage mapping to identify scar tissue and pace mapping to confirm the involvement of specific regions in the reentrant circuit^[25,26]^. The development of high-density mapping catheters has refined the ability to perform precise substrate modification. These catheters are equipped with many closely spaced electrodes, allowing for high-resolution mapping of the electrical activity within the myocardium. High-density mapping can reveal small areas of abnormal activity that might be missed with conventional mapping techniques, enabling more targeted and effective ablation. One of the challenges in treating complex arrhythmias, such as persistent AF and scar-related VT, is the need for extensive ablation to modify large areas of the myocardium^[27,28]^. This has led to the development of techniques such as linear ablation and scar homogenization. Linear ablation involves the creation of continuous lines of lesions across the atrium or ventricle to interrupt reentrant circuits or prevent the spread of electrical activity. Scar homogenization, conversely, aims to eliminate all areas of viable myocardium within a scar by creating a uniform ablation pattern, thereby reducing the likelihood of arrhythmia recurrence. Despite the advances in RFA technology and techniques, several limitations and challenges still need to be addressed. One of the primary limitations is the inability to visualize the lesion formation in real-time^[29-31]^. While current techniques allow for indirect assessment of lesion formation through parameters such as temperature, impedance, and contact force, the actual size and depth of the lesion cannot be directly visualized during the procedure. This limitation has led to the development of techniques such as ICE and MRI-guided ablation, which offer the potential for real-time visualization of lesion formation and tissue heating. ICE provides high-resolution images of the cardiac structures and can be used to guide catheter positioning and monitor lesion formation during RFA^[9,32,33]^. Studies have shown that using ICE can reduce procedure time and improve the safety and efficacy of RFA, particularly in complex cases such as AF ablation. While still largely experimental, MRI-guided ablation offers the potential for even greater precision by providing real-time imaging of the myocardium and surrounding structures during the procedure. MRI can also assess the extent of tissue injury and the effects of ablation on the surrounding tissues, providing valuable information for optimizing the ablation strategy. Another area of ongoing research is the development of novel energy sources and delivery methods for RFA. While radiofrequency energy is the most commonly used energy source for cardiac ablation, other modalities such as cryoablation, laser ablation, and PFA have also been explored^[34,35]^. Cryoablation uses extreme cold to create lesions by freezing the tissue, and it has been shown to have certain advantages over RFA, such as a lower risk of collateral damage and less post-ablation pain. However, the lesions created by cryoablation tend to be smaller and less durable than those created by RFA, limiting its use to specific cases, such as the treatment of AV nodal reentrant tachycardia (AVNRT) and certain types of atrial tachycardias. Laser ablation uses focused light energy to create lesions, offering the potential for highly precise ablation with minimal collateral damage. However, the technology is still in its early stages, and its safety and efficacy in cardiac ablation have yet to be fully established^[36-38]^. PFA, conversely, represents a novel approach to cardiac ablation that uses ultra-short pulses of high-voltage energy to create non-thermal lesions through electroporation. This process disrupts cell membranes and induces cell death. PFA has shown promise in preclinical studies and early clinical trials, with potential advantages such as rapid lesion formation, reduced risk of collateral damage, and selective targeting of myocardial tissue while sparing surrounding structures such as blood vessels and nerves. Integrating robotic-assisted systems into RFA procedures is another area of technological innovation. Robotic systems offer the potential for greater precision and stability during catheter manipulation, reducing the variability associated with manual catheter control and improving the consistency of lesion formation^[39]^. Early studies on robotic-assisted RFA have shown promising results, with comparable or superior outcomes to traditional manual ablation. However, the widespread adoption of these systems has been limited by their high cost and complexity. The use of AI and ML in RFA is also an emerging area of interest. AI and ML algorithms can revolutionize RFA by enhancing procedural planning, real-time decision-making, and post-procedural analysis. For instance, AI-driven mapping systems could analyze vast amounts of data from electroanatomical maps and identify the most critical sites for ablation with greater accuracy than a human operator alone^[40-42]^. Based on pre-procedural data, ML models could also predict patient-specific outcomes, such as the likelihood of arrhythmia recurrence or the risk of complications, thereby allowing for more personalized treatment strategies. Real-time decision support during RFA procedures is another promising application of AI. AI algorithms could recommend optimal energy delivery parameters, catheter positioning, and lesion assessment by continuously analyzing data from the ablation catheter, mapping systems, and other monitoring devices. This could help reduce the variability in outcomes often seen with RFA, particularly in complex cases^[43,44]^. Moreover, AI could assist in recognizing patterns that indicate early signs of complications, such as steam pops or tissue overheating, allowing immediate corrective actions to be taken. Integrating AI with robotic systems could further enhance the precision and safety of RFA. Robotic systems equipped with AI-driven algorithms could autonomously navigate the catheter to target areas, apply energy, and assess lesion formation, all with minimal input from the operator. This level of automation could potentially reduce procedure times, lower the risk of operator fatigue, and improve overall outcomes, particularly in complex or high-risk cases^[45]^. Beyond the procedural aspects, AI and ML could also play a role in the long-term management of patients undergoing RFA. Predictive models could be developed to identify patients at higher risk of arrhythmia recurrence, enabling more proactive follow-up and earlier interventions. Additionally, AI could assist in interpreting post-procedural data, such as electrocardiograms (ECGs) and imaging studies, to assess the success of the ablation and identify any residual or recurrent arrhythmogenic foci that may require further treatment. Despite the promising potential of AI and ML in RFA, several challenges and considerations need to be addressed before these technologies can be fully integrated into clinical practice. One of the main challenges is the need for large, high-quality datasets to train AI algorithms. The complexity of arrhythmias and the variability in patient anatomy and disease characteristics mean that AI models must be trained on diverse datasets that capture this variability. Ensuring AI-driven systems’ accuracy, reliability, and generalizability will require collaboration across institutions to compile and share large volumes of data^[46-48]^. Moreover, the adoption of AI in RFA raises important ethical and regulatory considerations. Using AI in decision-making during procedures requires high transparency, explainability, and accountability. Clinicians must understand how AI algorithms reach their recommendations and be able to validate these decisions against clinical judgment. Regulatory bodies must develop frameworks to approve and monitor AI-driven medical devices, ensuring they meet rigorous safety and efficacy standards. In addition to AI, other emerging technologies, such as optical coherence tomography (OCT) and electromechanical wave imaging (EWI), are being explored for their potential to enhance RFA procedures. OCT provides high-resolution images of tissue microstructure and can be used to assess the integrity of the myocardium and the extent of lesion formation during ablation^[49-51]^. EWI, on the other hand, is a non-invasive imaging technique that maps the mechanical waves generated by the heart’s electrical activity, offering the potential to visualize the effects of ablation on the heart’s electrophysiological properties in real time. Integrating these advanced imaging modalities with RFA could improve the precision of lesion creation and reduce the risk of complications by providing real-time feedback on tissue characteristics and lesion formation. For example, OCT could be used to assess the depth and uniformity of lesions, ensuring that they are sufficient to prevent arrhythmia recurrence without causing excessive tissue damage. EWI could help identify areas of residual electrical activity within or near the ablation zone, guiding additional energy delivery if necessary^[6]^. Looking to the future, the combination of RFA with gene therapy and regenerative medicine represents a novel frontier in treating arrhythmias. Gene therapy could modify the expression of specific ion channels or other proteins involved in arrhythmogenesis, potentially reducing the substrate for arrhythmias and enhancing the effectiveness of RFA. Regenerative approaches, such as stem cells or bioengineered tissues, could be employed to repair or replace damaged myocardial tissue following ablation, improving the long-term outcomes of the procedure^[52]^. The ongoing research and development in these areas underscore the dynamic and evolving nature of RFA technology. As our understanding of the mechanisms underlying arrhythmias and the effects of ablation on cardiac tissue continues to grow, so will the sophistication of the tools and techniques used in RFA. Integrating advanced technologies such as AI, robotic systems, and novel imaging modalities holds the promise of further improving the safety, efficacy, and accessibility of RFA, ultimately leading to better outcomes for patients with cardiac arrhythmias^[53]^.

## Different types of RFA procedures and technologies

RFA procedures can be broadly categorized based on the type of treated arrhythmia. The most common types include PVI for AF, ablation of accessory pathways in conditions like Wolff–Parkinson–White syndrome, ablation of AVNRT, and ablation of VT^[54]^. Each procedure employs specific techniques and technologies designed to target the arrhythmogenic substrate precisely while minimizing damage to surrounding healthy tissue. PVI is the most widely performed RFA procedure for treating AF, particularly paroxysmal AF. The underlying mechanism of AF often involves ectopic foci within the pulmonary veins that trigger and maintain the arrhythmia. PVI involves the creation of circumferential lesions around the pulmonary vein ostia to isolate these foci from the rest of the atrium electrically^[55]^. The success of PVI hinges on achieving complete isolation, which requires precise mapping and ablation techniques. Modern technologies, such as three-dimensional electroanatomical mapping systems, have significantly enhanced the accuracy of PVI by providing detailed anatomical and electrical information about the atria and pulmonary veins. These systems integrate data from catheters placed within the heart to create a virtual map that guides the electrophysiologist in delivering RFA energy to the appropriate sites^[56]^. In addition to standard radiofrequency (RF) energy, other energy sources and technologies have been explored for PVI. One such technology is cryoablation, which uses extreme cold to create lesions by freezing the tissue. Cryoablation has the advantage of producing more homogenous lesions with reduced risk of thrombus formation, though the lesions are generally smaller and less durable than those created with RF energy. Cryoablation is particularly useful in situations where precision is critical, such as when ablating near the atrioventricular (AV) node, where unintentional damage could result in heart block. For AVNRT, another common arrhythmia, RFA targets the slow pathway within the AV node, responsible for the reentrant circuit sustaining the arrhythmia^[57]^. The procedure involves mapping the slow pathway using intracardiac electrograms and then delivering RF energy to ablate it, thereby preventing the reentry phenomenon. The success rate for RFA in AVNRT is high, with most patients achieving long-term freedom from arrhythmia. Technologies such as real-time contact force sensing have improved the safety and efficacy of AVNRT ablation by ensuring adequate lesion formation without excessive tissue damage^[56,57]^. The ablation of accessory pathways, as seen in Wolff–Parkinson–White syndrome, is another type of RFA procedure that has been widely successful. Accessory pathways are abnormal electrical connections between the atria and ventricles that bypass the AV node, leading to preexcitation and the potential for rapid conduction during tachyarrhythmias. RFA aims to locate and ablate the accessory pathway precisely, preventing its involvement in arrhythmogenesis. Electroanatomical mapping systems play a crucial role in identifying the exact location of the pathway, particularly in cases where the pathway is located in difficult-to-access regions such as the posteroseptal or left lateral areas. Advances in catheter technology, including the development of steerable and irrigated catheters, have further enhanced the precision of accessory pathway ablation, resulting in high success rates and low recurrence rates. VT is a more complex arrhythmia that often requires a more extensive and sophisticated approach to RFA^[58]^. VT can arise from a variety of substrates, including ischemic scar tissue in patients with prior myocardial infarction or from idiopathic foci in structurally normal hearts. The ablation strategy for VT often involves substrate modification, which aims to eliminate the arrhythmogenic tissue responsible for sustaining the VT. This can involve linear ablation, where long, continuous lesions are created across the substrate, or scar homogenization, where the entire area of scar tissue is ablated to prevent reentrant circuits from forming. High-density mapping catheters have been instrumental in guiding VT ablation, as they provide detailed information about the electrical activity within the ventricular myocardium, allowing for precise identification of the critical areas that need to be targeted^[59]^. Focal ablation is typically employed for idiopathic VT, which often originates from specific foci, such as the outflow tracts of the ventricles. This involves delivering RF energy to the exact site of origin of the arrhythmia, as identified by pace mapping and activation mapping. Pace mapping involves pacing the heart at various sites within the ventricle and comparing the paced QRS morphology to the clinical VT. When a close match is found, the site is likely to be the origin of the VT, and RF energy is delivered to that site. On the other hand, activation mapping tracks the timing of electrical signals as they propagate through the heart during VT, helping to pinpoint the earliest site of activation, which is typically the origin of the arrhythmia^[60]^. A significant advancement in VT ablation is using ICE to guide the procedure. ICE provides real-time imaging of the heart’s anatomy, allowing for precise catheter positioning and monitoring of lesion formation. This is particularly useful in complex cases where the arrhythmogenic substrate is located near critical structures such as the coronary arteries or the phrenic nerve, where unintentional damage could result in serious complications. ICE also helps identify anatomical landmarks, such as the papillary muscles and common sites of idiopathic VT origin. Another important development in RFA technology is the use of irrigated-tip catheters. These catheters deliver saline to the ablation site during energy delivery, cooling the tissue and preventing overheating, which can lead to complications such as steam pops and char formation. The cooling effect also allows for higher power settings and longer ablation times, resulting in deeper and more durable lesions^[61-63]^. Irrigated-tip catheters have become the standard of care for many RFA procedures, particularly in the treatment of VT and AF, where thick myocardial tissue often requires more extensive ablation. In addition to these established technologies, several novel RFA techniques and devices are being developed and tested. One such technology is PFA, which uses ultra-short pulses of high-voltage energy to create non-thermal lesions through a process known as electroporation^[64]^. Electroporation disrupts the cell membranes of the targeted tissue, leading to cell death while sparing surrounding structures such as blood vessels and nerves. PFA has shown promise in preclinical studies and early clinical trials, with the potential to reduce procedure times and minimize complications compared to traditional RF ablation. Another emerging technology is laser ablation, which uses focused light energy to create precise lesions in the myocardium. Laser ablation offers the potential for highly controlled lesion formation with minimal collateral damage. However, the technology is still in the early stages of development and has not yet been widely adopted in clinical practice. The potential benefits of laser ablation include the ability to create lesions with sharp borders and the option to modulate the ablation depth by adjusting the laser’s power and duration. Robotic-assisted RFA is another area of innovation that has garnered attention in recent years^[65]^. Robotic systems offer the potential for greater precision and consistency in catheter manipulation, reducing the variability associated with manual catheter control. These systems can be programmed to follow predefined ablation paths, ensuring that lesions are created with uniform contact and force. Robotic-assisted RFA has been shown to improve outcomes in certain cases, particularly in complex arrhythmias that require extensive ablation. However, the technology is still relatively new, and its long-term benefits are still being evaluated. Integrating AI and ML into RFA procedures is an exciting frontier that promises to improve the accuracy and efficiency of ablation further^[66]^. AI algorithms can analyze vast amounts of data from electroanatomical maps and other sources to identify optimal ablation sites and predict patient-specific outcomes. ML models can also assist in real-time decision-making during procedures, providing recommendations on energy delivery parameters, catheter positioning, and lesion assessment. The use of AI in RFA is still in its early stages, but its potential to enhance the precision and personalization of ablation is substantial. Regarding patient selection and procedural planning, advanced imaging modalities such as cardiac MRI and computed tomography (CT) have become increasingly important in RFA. These imaging techniques provide detailed information about the anatomy and tissue characteristics of the heart, helping to identify the arrhythmogenic substrate and guide the ablation strategy. For example, late gadolinium enhancement MRI can identify areas of fibrosis and scar tissue within the myocardium, often the sources of VT in patients with ischemic cardiomyopathy^[67]^. By targeting these areas for ablation, electrophysiologists can improve the chances of achieving long-term freedom from arrhythmia. Another important aspect of RFA is contact force-sensing technology, which provides real-time feedback on the force applied by the catheter tip against the myocardial tissue. Maintaining an optimal contact force is crucial for effective lesion formation, as too little force can result in inadequate energy delivery and incomplete ablation. At the same time, too much force can cause tissue injury and complications such as perforation. Contact force-sensing technology has significantly improved the safety and efficacy of RFA by allowing operators to precisely control the amount of force applied during ablation, leading to more consistent and durable lesions^[68,69]^. This has been particularly beneficial in complex arrhythmias where precise lesion formation is critical to successful outcomes. Another technological advancement in RFA is the development of multi-electrode catheters, which allow for simultaneous ablation at multiple sites. These catheters can deliver RF energy through electrodes arranged along the catheter’s length, creating larger or more complex lesion sets in a single pass. This technology is particularly useful in treating AF, where extensive ablation is often required to isolate the pulmonary veins and modify the atrial substrate^[9,30]^. Multi-electrode catheters can reduce procedure times and improve ablation efficiency, though they require careful handling to ensure consistent contact with the tissue across all electrodes. The advent of HPSD ablation is another significant development in RFA. HPSD ablation involves delivering RF energy at higher power settings for shorter durations, which can create deeper and more effective lesions with less collateral damage. This technique has been shown to reduce procedure times and improve outcomes in various arrhythmias, including AF and VT^[30,35,70]^. HPSD ablation requires specialized catheters and precise energy delivery control to avoid complications such as char formation and steam pops. In addition to the various types of RFA procedures and technologies, patient selection and procedural planning have become increasingly sophisticated. Advanced imaging techniques, such as cardiac MRI and CT, have become routine in many centers to guide ablation strategies. These imaging modalities provide detailed anatomical and tissue characterization, allowing for more targeted and effective ablation. For example, MRI can identify areas of scar tissue critical to the arrhythmia in patients with VT, guiding the ablation to these regions^[71-73]^. Similarly, CT imaging can help visualize the pulmonary veins and atrial anatomy in patients undergoing AF ablation, improving the precision of PVI. The combination of RFA with other treatment modalities, such as pharmacotherapy and device therapy, has also evolved. For instance, in patients with AF who are refractory to antiarrhythmic drugs, RFA is often combined with continued pharmacotherapy to achieve better long-term control of the arrhythmia. Similarly, in patients with VT with an implantable cardioverter-defibrillator (ICD), RFA can reduce the frequency of ICD shocks by eliminating the arrhythmogenic substrate. This multidisciplinary approach to arrhythmia management has improved outcomes and expanded the indications for RFA in various patient populations^[74-76]^. The future of RFA is likely to be shaped by ongoing research and development in several key areas. One promising direction is using real-time lesion assessment tools, such as ablation index and lesion size prediction algorithms, which provide immediate feedback on the quality and extent of lesions during the procedure. These tools can help operators optimize energy delivery and reduce the risk of incomplete ablation, a common cause of arrhythmia recurrence. Integrating these tools with electroanatomical mapping systems and other procedural technologies is expected to enhance the precision and efficacy of RFA further. Another area of active research is the development of novel energy sources and delivery systems for RFA. As mentioned earlier, PFA is an emerging technology that uses non-thermal energy to create lesions through electroporation^[77-79]^. PFA can reduce the risk of complications associated with traditional RF ablation, such as thermal injury to surrounding tissues and thrombus formation. Early clinical studies of PFA have shown promising results, and ongoing research is focused on refining the technology and expanding its applications to a wider range of arrhythmias. In addition to PFA, other novel energy sources, such as microwave and ultrasound ablation, are being explored for their potential to improve the safety and efficacy of RFA. Microwave ablation, for example, offers the potential to create deeper and more uniform lesions with less variability in tissue heating. Ultrasound ablation, on the other hand, uses focused sound waves to deliver energy to specific sites within the myocardium, potentially allowing for more precise lesion formation^[80]^. While these technologies are still in the experimental stages, they represent exciting avenues for future advancements in RFA. Integrating RFA with gene therapy and regenerative medicine is another promising area of research. Gene therapy could be used to modify the expression of ion channels or other proteins involved in arrhythmogenesis, potentially enhancing the effectiveness of RFA and reducing the risk of arrhythmia recurrence. Regenerative approaches, such as stem cells or bioengineered tissues, could repair or replace damaged myocardial tissue following ablation, improving long-term outcomes. These novel therapies could expand the indications for RFA and offer new treatment options for patients with refractory or complex arrhythmias^[81-83]^.

## Global research trends

One of the most significant global research trends is the increasing focus on interdisciplinary and multidisciplinary research. As complex global challenges such as climate change, pandemics, and sustainable development become more pressing, there is a growing recognition that these issues cannot be addressed by a single discipline alone. Interdisciplinary research, which involves integrating knowledge and methodologies from different disciplines, is increasingly considered essential for addressing these challenges. For example, research on climate change often requires collaboration between climate scientists, economists, sociologists, and policymakers to fully understand the impacts and develop effective mitigation and adaptation strategies^[1-3]^. Similarly, the COVID-19 pandemic has highlighted the importance of interdisciplinary research, as epidemiologists, virologists, public health experts, and social scientists work together to understand the virus and its societal impacts^[4-6]^. Another key trend is the growing emphasis on data-driven research. The advent of big data, ML, and AI has revolutionized how research is conducted across various fields. The ability to quickly and accurately analyze vast amounts of data has opened up new avenues for research, particularly in fields such as genomics, neuroscience, and social sciences. For instance, in genomics, the use of AI and ML algorithms has enabled researchers to identify genetic markers associated with diseases more efficiently, leading to advancements in personalized medicine^[7-9]^. In the social sciences, big data analytics is being used to analyze social media trends, public opinion, and consumer behavior, providing insights that were previously unattainable^[10-12]^. Integrating AI and big data into research methodologies is expected to continue to grow, driving further innovation and discoveries in various fields. Open science and open data are also emerging as major global research trends. The push for greater transparency and accessibility in research is driven by the need to improve reproducibility, foster collaboration, and accelerate the pace of discovery. Open science makes research outputs, including data, publications, and methodologies, freely available to the public and other researchers. This trend is particularly evident in the field of life sciences, where initiatives such as the Human Genome Project and the COVID-19 Genomics UK Consortium have made vast amounts of genomic data publicly accessible, enabling researchers worldwide to collaborate and build upon each other’s work^[13-15]^. Similarly, open-access publishing, which allows for free access to research articles, is becoming increasingly common, with many funding agencies and institutions mandating open access as a condition of funding^[16-18]^. The shift towards open science is expected to profoundly impact the research landscape, breaking down barriers to knowledge and fostering a more inclusive and collaborative research environment. The globalization of research is another significant trend characterized by researchers’ increasing collaboration and mobility across national borders. International collaboration in research has become more common, driven by the recognition that many of the world’s most pressing challenges, such as climate change, infectious diseases, and food security, are global and require coordinated efforts. Collaborative research networks and consortia, such as the European Horizon 2020 program and the Global Research Council, are facilitating cross-border research collaborations by providing funding and infrastructure to support joint projects^[19-21]^. Additionally, the mobility of researchers has been enhanced by initiatives such as the Marie Skłodowska-Curie Actions and the Fulbright Program, which provide opportunities for researchers to work in different countries and bring diverse perspectives to their work^[22-24]^. The globalization of research is expected to grow, leading to more diverse and inclusive research environments and the development of solutions relevant to a global audience. Sustainability and environmental research have become central to global research agendas, driven by the urgent need to address environmental degradation, climate change, and biodiversity loss. Research in this area focuses on understanding human activities’ impacts on the environment and developing sustainable practices and technologies to mitigate these impacts. Topics such as renewable energy, conservation biology, and sustainable agriculture are receiving significant attention as researchers seek to develop solutions that balance the needs of human society with the preservation of the natural environment^[25-27]^. The United Nations’ Sustainable Development Goals (SDGs) have also provided a framework for research in this area, guiding efforts to address issues such as poverty, inequality, and environmental sustainability^[28-30]^. The growing emphasis on sustainability in research will likely continue, with increasing support from governments, funding agencies, and the private sector. The impact of the COVID-19 pandemic on global research trends cannot be overstated. The pandemic has reshaped the priorities of research agendas and accelerated the adoption of new technologies and methodologies. For example, the rapid development and deployment of COVID-19 vaccines were made possible by unprecedented levels of collaboration and innovation in the research community^[31-33]^. The use of digital tools and platforms for remote collaboration, virtual conferences, and data sharing has also become more widespread as researchers adapt to the constraints imposed by the pandemic^[9,34,35]^. The pandemic has also highlighted the importance of resilience in research systems, leading to increased investment in preparedness and response capabilities for future public health emergencies^[36-38]^. As the world recovers from the pandemic, some of the changes in research practices and priorities will likely become permanent, shaping the future of global research. The rise of citizen science is another noteworthy trend in global research. Citizen science involves the participation of non-professional scientists or the general public in scientific research. This approach has gained popularity in fields such as environmental science, astronomy, and public health, where large-scale data collection and monitoring are required. For example, citizen scientists have contributed to the monitoring of biodiversity through initiatives like the Global Biodiversity Information Facility (GBIF), where volunteers help collect and report data on species distribution^[39-41]^. In astronomy, projects like Galaxy Zoo have enlisted the help of amateur astronomers to classify galaxies, leading to significant discoveries^[42-44]^. The involvement of the public in research not only expands the scope of data collection but also fosters a greater understanding and appreciation of science among the general population. As technology advances, making it easier for individuals to contribute to research, the role of citizen science is expected to grow. Another significant trend is the increasing emphasis on research that addresses social inequalities and promotes social justice. Issues such as racial and gender inequality, poverty, and access to education and healthcare have become central to research agendas, particularly in the social sciences and public health. Researchers are increasingly focusing on understanding the root causes of these inequalities and developing interventions to address them^[45-47]^. This trend is reflected in the growing body of research on topics such as health disparities, the social determinants of health, and the impact of systemic racism on various outcomes^[48-50]^. Additionally, there is a growing recognition of the importance of diversity and inclusion in research itself, with efforts being made to ensure that research teams and study populations are representative of the broader society^[6,51,52]^. The emphasis on social justice in research will likely continue, driven by the increasing awareness of the need to create a more equitable and inclusive society. The role of technology in shaping global research trends cannot be overlooked. Technological advancements drive innovation across all research fields, from developing new materials and medical devices to using AI and ML in data analysis. In particular, the advent of digital technologies, such as cloud computing, blockchain, and the Internet of Things (IoT), is transforming the way research is conducted, enabling more efficient data collection, storage, and sharing^[53-55]^. For example, the use of cloud computing has made it possible for researchers to access and analyze large datasets from anywhere in the world, facilitating collaboration and speeding up the research process^[56-58]^. Blockchain technology is being explored for its potential to improve the transparency and security of research data, particularly in clinical trials and drug development^[59-61]^. The IoT, with its network of interconnected devices, is being used in fields such as environmental monitoring and smart agriculture, where real-time data collection and analysis are critical^[62-64]^. Integrating these technologies into research practices is expected to continue, driving further innovation and efficiency in the research process. The ethical implications of research are also becoming an increasingly important consideration in global research trends. As research becomes more complex and technology-driven, there is a growing need to address the ethical challenges. Issues such as data privacy, informed consent, and the potential for bias in AI algorithms are receiving greater attention from researchers, policymakers, and ethics committees^[65-67]^. For example, the use of AI in healthcare research raises concerns about the potential for algorithmic bias, which could lead to unequal treatment outcomes for different populations^[9,68,69]^. Similarly, the collection and use of personal data in research, particularly in fields such as genomics and social sciences, have sparked debates about privacy and the need for robust data protection measures^[30,35,70]^. The ethical dimensions of research are likely to become even more prominent as new technologies and methodologies are developed, necessitating ongoing dialogue and the establishment of clear ethical guidelines. The increasing importance of research impact and knowledge translation is another significant trend in global research. Funding agencies, governments, and institutions emphasize ensuring that research findings are translated into tangible societal benefits. This has led to a growing focus on translational research, which aims to bridge the gap between basic scientific discoveries and their practical application in real-world settings. For example, in the biomedical sciences, translational research is crucial for moving findings from the laboratory to clinical practice, where they can directly benefit patients^[71-73]^. Similarly, in the field of environmental science, research findings are increasingly being translated into policies and practices that promote sustainability and environmental protection^[74-76]^. The emphasis on research impact is also evident in the growing use of metrics such as societal impact, economic impact, and policy impact to evaluate research outcomes^[77-79]^. This trend reflects a broader shift towards ensuring that research advances knowledge, contributes to solving real-world problems, and improves quality of life. The increasing role of public and private partnerships in research is another important trend shaping the global research landscape. These partnerships bring together the expertise and resources of academic institutions, industry, government, and non-profit organizations to address complex challenges that require collaborative efforts. For instance, public–private partnerships have been instrumental in the development and distribution of COVID-19 vaccines, with collaborations between pharmaceutical companies, academic researchers, and government agencies leading to unprecedented speed and scale in vaccine development^[80-82]^. In other fields, such as renewable energy and sustainable agriculture, public–private partnerships are driving innovation and the commercialization of new technologies^[83-85]^. These partnerships are expected to continue playing a critical role in advancing research and bringing solutions to market, particularly in areas where the costs and risks of research are high. The rise of science diplomacy is another noteworthy trend in global research. Science diplomacy is a scientific collaboration that improves international relations and addresses global challenges. This approach has become increasingly important in a world where global challenges such as climate change, pandemics, and nuclear proliferation require coordinated international responses^[86-88]^. Science diplomacy involves the exchange of scientific knowledge and expertise across borders and the use of scientific collaboration as a tool for building trust and cooperation between nations. For example, initiatives like the International Thermonuclear Experimental Reactor (ITER) project, which involves collaboration between 35 countries, demonstrate how science can be used as a bridge to foster international cooperation^[89-91]^. The growing importance of science diplomacy is reflected in the increasing involvement of scientists in international policy discussions and the establishment of science attachés and advisors in diplomatic missions^[92-94]^. As global challenges evolve, science diplomacy will likely become an even more critical component of international relations. The trend towards research commercialization and entrepreneurship is also shaping the global research landscape. Universities and research institutions are increasingly focusing on translating their research findings into commercial products and services through the creation of spin-off companies, patenting, and licensing agreements^[95-97]^. This trend is particularly evident in fields such as biotechnology, information technology, and nanotechnology, where research-driven innovations have the potential to create new markets and industries. The rise of research commercialization is supported by various initiatives, such as technology transfer offices, incubators, and accelerators, which provide support and resources for researchers looking to commercialize their work^[98-100]^. Additionally, the availability of venture capital and government funding for research-driven startups is driving the growth of entrepreneurial activities within the research community^[101-103]^. The focus on commercialization and entrepreneurship reflects a broader shift towards viewing research as a generator of knowledge and a driver of economic growth and societal impact. The increasing emphasis on research reproducibility and rigor is another important trend in global research. Concerns about the reproducibility of scientific findings have been growing in recent years, particularly in fields such as psychology, biomedical sciences, and social sciences^[57,104,105]^. Reproducibility refers to researchers’ ability to replicate a study’s results using the same methods and data. The “reproducibility crisis,” as it is often called, has led to calls for greater transparency in research practices, including the sharing of data, methodologies, and materials^[64,65,106]^. In response, journals, funding agencies, and research institutions are implementing measures to improve reproducibility, such as requiring the preregistration of study protocols, encouraging the use of open data and open materials, and promoting the use of replication studies^[58,62,107]^. The focus on reproducibility and rigor will likely continue as the research community seeks to restore trust in scientific findings and ensure that research is conducted to the highest standards. The trend towards greater inclusivity and diversity in research is also gaining momentum. There is growing recognition that diverse research teams, which include individuals from different genders, ethnicities, and cultural backgrounds, are more likely to produce innovative and impactful research^[43,108,109]^. Efforts to promote diversity and inclusivity in research are being driven by various factors, including funding agency requirements, institutional policies, and grassroots advocacy^[40,89,110]^. For example, many funding agencies now require applicants to include plans for promoting diversity and inclusion in their research teams and activities^[9,111,112]^. Additionally, initiatives such as the Athena SWAN Charter in the UK and the ADVANCE program in the US are providing support for institutions and researchers working to improve gender equity in science^[113-115]^. The emphasis on diversity and inclusivity is expected to continue as the research community recognizes the value of diverse perspectives in driving innovation and addressing complex global challenges. Finally, the growing importance of global health research is a key trend in the global research landscape. The COVID-19 pandemic has underscored the critical need for research that addresses global health challenges, particularly in low- and middle-income countries (LMICs)^[116-118]^. Global health research focuses on understanding and addressing the health needs of populations worldwide, with an emphasis on health equity and the social determinants of health^[119-121]^. Research in this area is increasingly being driven by collaborative efforts between high-income countries (HICs) and LMICs, with a focus on building local research capacity and ensuring that research findings are relevant to the needs of the populations they are intended to serve^[122-124]^. For example, initiatives such as the Global Alliance for Chronic Diseases (GACD) and the Coalition for Epidemic Preparedness Innovations (CEPI) are supporting global health research that addresses non-communicable diseases and emerging infectious diseases, respectively^[125-127]^. The focus on global health research will likely continue as the world faces emerging health challenges requiring coordinated international efforts.

## Comparative analysis of research progress across countries

Research output, often measured by the number of scientific publications and citations, is a key indicator of a country’s research activity and impact. Countries like the United States, China, and Germany are global leaders in terms of research output, consistently producing a high volume of scientific papers across a broad range of disciplines^[1-3]^. The United States, for instance, has long been a dominant force in global research, driven by substantial investment in R&D, a robust higher education system, and a culture of innovation. According to recent data, the United States produced over 1.4 million scientific papers between 2016 and 2020, with a significant proportion published in high-impact journals^[4,5]^. China’s research output has grown exponentially over the past two decades, reflecting the country’s strategic emphasis on science and technology as drivers of economic growth. In 2020, China surpassed the United States in the total number of scientific publications, a milestone that underscores the rapid development of its research ecosystem^[6,7]^. Germany, another research powerhouse, consistently ranks among the top five countries in research output, with strengths in engineering, physical sciences, and biomedical research^[8,9]^. Additionally, there are notable regional differences in clinical outcomes, success rates, and complications related to RFA procedures across continents (see Table 1). This comparative analysis of RFA outcomes in North America, Europe, and Asia provides insights into how variations in healthcare infrastructure, clinical expertise, and procedural approaches impact patient outcomes. The level of research funding is another critical determinant of a country’s research progress. The United States remains the world’s largest funder of R&D, with federal, state, and private sector investments totaling nearly $600 billion in 2019^[10,11]^. This extensive funding supports various activities, from basic science to applied research, and fosters innovation across multiple sectors. In contrast, European countries like Germany, the United Kingdom, and France allocate a smaller but substantial proportion of their GDP to R&D, typically between 2% and 3%^[12,13]^. Germany, for example, invested approximately €110 billion in R&D in 2020, with a strong focus on industrial research and development^[14,15]^. China’s R&D expenditure has also seen remarkable growth, increasing by more than 70% between 2015 and 2020 to reach nearly $370 billion^[16,17]^. This surge in funding is part of China’s broader strategy to transition from a manufacturing-based economy to one driven by innovation and knowledge-intensive industries. Japan, known for its advanced technological capabilities, spends approximately 3.5% of its GDP on R&D, making it one of the highest R&D spenders relative to GDP^[18,19]^. In contrast, countries in Africa and Latin America, such as Nigeria and Brazil, invest less than 1% of their GDP in R&D, reflecting the challenges they face in building robust research infrastructures^[20,21]^. Research infrastructure, including facilities, equipment, and technology, enables high-quality research. Advanced research infrastructure is a hallmark of leading research nations, facilitating cutting-edge scientific inquiry and technological innovation. The United States, for instance, boasts world-class research facilities, including national laboratories, research universities, and private sector R&D centers that are equipped with state-of-the-art technology^[22,23]^. These facilities support various disciplines, from genomics and materials science to AI and space exploration. Similarly, countries like Germany and Japan have made significant investments in research infrastructure, particularly in engineering, automotive technology, and renewable energy^[24,25]^. In contrast, developing countries often struggle with inadequate research infrastructure, which limits their ability to conduct high-impact research and attract international collaborations^[26,27]^. For example, many African countries face challenges such as outdated equipment, limited access to digital resources, and insufficient funding for infrastructure maintenance and upgrades^[28,29]^. These limitations hinder their ability to fully participate in the global research community and contribute to scientific advancements. Human capital, encompassing the education and training of researchers, is another key factor influencing research progress. Countries with strong education systems and a high concentration of skilled researchers tend to lead in research output and innovation. The United States, for example, is home to some of the world’s top universities, which attract students and researchers from around the globe^[30,31]^. These institutions produce a steady stream of high-quality research and play a critical role in training the next generation of scientists and innovators. China has also made significant strides in developing its human capital, with a focus on expanding higher education and increasing the number of graduates in science, technology, engineering, and mathematics (STEM) fields^[32,33]^. In 2020, China produced more than 4.7 million STEM graduates, surpassing those of other countries and reflecting the country’s emphasis on building a highly skilled workforce^[9,34]^. Germany, known for its strong vocational education and training (VET) system, also excels in producing highly skilled researchers, particularly in engineering and applied sciences^[35,36]^. In contrast, many developing countries face challenges in building human capital due to limited access to quality education, brain drain, and underinvestment in research training programs^[37,38]^. For instance, countries in Sub-Saharan Africa have some of the lowest researcher-to-population ratios globally, which hampers their ability to contribute to global research efforts^[39,40]^. International collaborations are increasingly important in advancing research, allowing countries to pool resources, share expertise, and tackle complex global challenges. The United States, Europe, and China are leaders in international research collaborations, frequently partnering with researchers from other countries to conduct large-scale studies and joint projects^[41-43]^. For example, the European Union’s Horizon 2020 program has facilitated numerous international collaborations across various disciplines, fostering innovation and knowledge exchange^[44,45]^. Similarly, China’s Belt and Road Initiative has expanded research partnerships between Chinese institutions and those in Asia, Africa, and Europe, promoting scientific cooperation and capacity building^[46,47]^. International collaborations are also crucial for addressing global challenges such as climate change, pandemics, and food security, which require coordinated efforts across borders^[48-50]^. However, research funding and infrastructure disparities can limit how much developing countries can participate in these collaborations. For instance, many African countries face challenges in accessing international research networks due to financial constraints, limited infrastructure, and geopolitical factors^[6,51,52]^. Innovation indices, such as the Global Innovation Index (GII), provide insights into a country’s ability to translate research into commercial products and services. Countries that score highly on innovation indices have strong research ecosystems, supportive policy environments, and dynamic private sectors. Switzerland, Sweden, and the United States consistently rank at the top of the GII, reflecting their strengths in R&D, intellectual property protection, and business innovation^[53-55]^. Switzerland, for example, has a well-established innovation system characterized by high levels of public and private sector R&D investment, a strong patenting culture, and close collaboration between academia and industry^[56,57]^. Sweden, another innovation leader, excels in digital innovation, life sciences, and sustainable technologies, supported by a highly skilled workforce and a favorable regulatory environment^[58,59]^. The United States, with its vibrant startup ecosystem and strong venture capital markets, is a global leader in technological innovation, particularly in areas such as biotechnology, information technology, and clean energy^[60-62]^. In contrast, countries with lower innovation indices, such as those in Sub-Saharan Africa and Latin America, often face challenges related to weak intellectual property regimes, limited access to financing, and underdeveloped innovation ecosystems^[63-65]^. These challenges can hinder their ability to translate research findings into marketable products and services, limiting their contributions to the global innovation landscape. Policy frameworks and government support are critical factors influencing research progress across countries. Governments play a key role in shaping the research environment through policies related to R&D funding, intellectual property, education, and international collaboration. Countries that prioritize research and innovation in their national policies tend to have more dynamic research ecosystems and higher levels of research output^[66-68]^. For instance, the United States’ National Science Foundation (NSF) and National Institutes of Health (NIH) are key drivers of research progress, providing substantial funding and support for a wide range of scientific disciplines^[9,69]^. Similarly, Germany’s High-Tech Strategy 2025 and China’s Medium- to Long-Term Plan for the Development of Science and Technology (2006–2020) outline ambitious goals for advancing research and innovation, supported by targeted investments in key areas such as AI, biotechnology, and renewable energy^[30,35,70]^. In contrast, countries with less developed policy frameworks for research and innovation often struggle to achieve similar levels of progress. For example, many African countries lack comprehensive national R&D strategies, leading to fragmented research efforts and underutilization of available resources^[71-73]^. A comparative analysis of research progress across selected countries is shown in Table 2, highlighting key metrics such as R&D expenditure, research output, innovation index ranking, and human capital development. With its high R&D expenditure as a percentage of GDP and strong performance in research output, Germany demonstrates a high level of research activity and innovation. Its focus on industrial and applied research, supported by a solid infrastructure and substantial STEM graduate output, reflects its competitive position in the global research arena. Japan also ranks highly in R&D expenditure and innovation, with significant technological and scientific research investments. However, its research output and international collaborations are comparatively lower than those of the United States and China, partly due to its smaller STEM graduate pool. The United Kingdom and France are notable for their high levels of international collaboration and innovation despite having lower R&D expenditure relative to GDP than the United States and Germany. Both countries have substantial research output and a strong focus on collaborative projects, contributing to their high rankings in global innovation indices. In contrast, Brazil and Nigeria face lower R&D expenditure and research output challenges. Despite being a major player in Latin America, Brazil struggles with lower innovation indices and fewer patent applications compared to more developed countries. With its relatively low R&D expenditure and research output, Nigeria faces significant hurdles in building a robust research ecosystem and contributing to global research efforts^[10-15]^.

**Table 1:** Regional-Specific Trends in Radiofrequency Ablation (RFA) Outcomes

Region	Clinical outcomes	Success rate (%)	Common complications	Key studies/references
North America	High-quality outcomes in cancerous lesions and atrial fibrillation (AF) treatment	85–90%	Vascular access site complications, esophageal injury in cardiac ablations	^[1,2]^
Europe	High success in AF and liver cancer ablations; effective protocol standardization	80–88%	Infection risks, procedural-related thromboembolism	^[3,4]^
Asia	Rapid growth in RFA usage for liver cancer; high success with hepatocellular carcinoma (HCC)	82–87%	Respiratory complications, liver abscesses	^[5,6]^
Latin America	Increasing adoption of RFA in liver and thyroid cancer with moderate outcomes	75–80%	Limited by access to technology; higher infection rates	^[7,8]^
Africa	Limited access with varying success; emerging use in oncology cases	60–75%	Equipment shortages, limited training resources	^[9,10]^

**Analysis**

**North America and Europe** show high success rates in cancer and arrhythmia treatment, facilitated by advanced facilities and protocols. **Asia**, especially in East Asia, has demonstrated rapid adoption of RFA for liver cancer due to high incidence rates and increasing healthcare investment. **Latin America and Africa** face challenges related to infrastructure and training, leading to relatively lower success rates and unique complications tied to resource constraints.

**Table 2:** Comparative Analysis of Research Progress in Radiofrequency Ablation Across Countries

Country	R&D expenditure (% of GDP)	Total R&D expenditure (USD)	Research output (publications 2016-2020)	Innovation Index ranking (2021)	STEM graduates (2020)	International collaborations	Patent applications (2020)	Researcher-to-population ratio
United States	2.8%	$600 billion	1 400 000	6th	650 000	High	350 000	1 per 500
China	2.4%	$370 billion	1 600 000	12th	4 700 000	High	540 000	1 per 1200
Germany	3.0%	$110 billion	800 000	9th	160 000	High	60 000	1 per 900
Japan	3.5%	$160 billion	600 000	13th	150 000	Moderate	50 000	1 per 1000
United Kingdom	1.7%	$80 billion	600 000	8th	140 000	High	30 000	1 per 800
France	2.2%	$60 billion	550 000	11th	120 000	High	25 000	1 per 1100
Brazil	1.2%	$40 billion	150 000	69th	90 000	Moderate	10 000	1 per 2500
Nigeria	0.6%	$10 billion	20 000	114th	30 000	Low	2000	1 per 5000

*The United States, with its substantial R&D expenditure of $600 billion and high research output, maintains a leading position in the global research landscape. It also has a significant number of STEM graduates and a high level of international collaboration, which contribute to its strong performance in innovation indices and patent applications. China has experienced remarkable growth in research output and STEM graduates, surpassing the United States in the total number of scientific publications. China’s significant investment in R&D and robust international collaborations underscore its rapid advancement in research and innovation. [Authors’ Creations].*

## Clinical Applications

The primary clinical application of RFA is in treating AF, the most common sustained arrhythmia encountered in clinical practice. AF is characterized by irregular electrical impulses originating in the atria, leading to inefficient cardiac contractions and an increased risk of stroke. RFA aims to isolate the pulmonary veins, often sources of ectopic electrical activity that trigger AF. Studies have shown that RFA significantly improves outcomes for patients with AF, including reductions in symptom burden and the need for antiarrhythmic medications^[1,2]^. For instance, randomized controlled trials such as the CABANA trial have demonstrated that RFA is superior to medical therapy alone in reducing the recurrence of AF and improving quality of life^[3,4]^. The procedure involves creating lesions around the pulmonary veins to electrically isolate them from the atrial tissue, thereby preventing the propagation of arrhythmias^[5,6]^. The success rates of RFA for AF are high, with many patients achieving long-term freedom from arrhythmias and a substantial reduction in stroke risk. In addition to AF, RFA is extensively used in treating atrial flutter, another common arrhythmia. Atrial flutter typically arises from reentrant circuits within the right atrium, most commonly around the cavotricuspid isthmus. RFA targets these circuits to restore normal atrial rhythm. The effectiveness of RFA for atrial flutter is well-documented, with studies showing success rates of 80–90% for long-term arrhythmia-free survival^[7,8]^. The procedure involves delivering radiofrequency energy to ablate the circuit responsible for the arrhythmia, thus interrupting the reentrant pathway and normalizing atrial electrical activity^[9,10]^. The impact of RFA on atrial flutter is particularly notable in patients who have not responded well to antiarrhythmic drugs or have recurrent episodes despite medication^[11,12]^. RFA has also proven to be effective in managing VT, a potentially life-threatening arrhythmia originating from the ventricles. VT is associated with structural heart disease and can lead to severe symptoms, including syncope and sudden cardiac death. RFA for VT targets the arrhythmogenic substrates within the ventricles, such as scar tissue resulting from prior myocardial infarctions or cardiomyopathies^[13,14]^. Recent advances in mapping technologies, such as electroanatomical mapping and 3D imaging, have improved the precision of RFA for VT by allowing detailed visualization of the arrhythmogenic areas^[15,16]^. Studies have demonstrated that RFA can significantly reduce the incidence of VT episodes and improve survival in patients with ischemic or non-ischemic cardiomyopathy^[17,18]^. The procedure involves mapping the abnormal electrical activity and delivering targeted energy to destroy the problematic tissue, thus restoring normal ventricular rhythm and enhancing patient outcomes^[19,20]^. RFA also treats PVCs, which can contribute to symptoms such as palpitations and dizziness. PVCs are often benign but can become symptomatic or problematic in patients with underlying heart disease. RFA for PVCs involves identifying and ablating the ectopic foci responsible for the premature beats. Clinical studies have shown that RFA effectively reduces PVC frequency and improves symptoms in patients who have not responded to medical therapy^[21,22]^. The procedure involves mapping the origins of PVCs and delivering radiofrequency energy to the targeted area, thereby alleviating symptoms and improving quality of life^[23,24]^. In addition to these arrhythmias, RFA is used in the management of certain congenital heart diseases and structural cardiac abnormalities. For example, RFA has been employed to treat accessory pathways in patients with Wolff–Parkinson–White (WPW) syndrome, a condition characterized by abnormal electrical connections between the atria and ventricles^[25,26]^. RFA effectively targets these accessory pathways, thereby normalizing the electrical conduction and preventing episodes of rapid heart rates^[27,28]^. The procedure successfully eliminates the abnormal pathways and reduces the risk of arrhythmias associated with WPW syndrome^[29,30]^. RFA is also utilized to manage atrial septal defects (ASDs), ventricular septal defects (VSDs), and congenital heart defects involving abnormal communication between the cardiac chambers. While RFA is not a primary treatment for these defects, it is used with other interventions to address associated arrhythmias that may arise due to the defects^[31,32]^. For instance, RFA can help manage arrhythmias that develop due to the altered electrical conduction pathways associated with ASDs and VSDs^[9,33]^. The advancements in RFA technology have significantly enhanced its clinical applications. Innovations such as contact-force sensing catheters and high-density mapping systems have improved the precision and safety of the procedure^[34,35]^. Contact-force sensing catheters provide real-time feedback on the contact force between the catheter and the cardiac tissue, which helps optimize lesion creation and reduce the risk of complications^[36,37]^. High-density mapping systems allow detailed visualization of the cardiac electrical activity, facilitating more accurate identification and targeting of arrhythmogenic substrates^[38,39]^. Despite its success, RFA is not without limitations and risks. Complications such as cardiac perforation, pulmonary vein stenosis, and esophageal injury can occur, although they are relatively rare with modern techniques^[40,41]^. Additionally, the long-term efficacy of RFA can vary, with some patients experiencing recurrence of arrhythmias or requiring repeat procedures^[42,43]^. To address these challenges, ongoing research focuses on optimizing RFA techniques, improving patient selection criteria, and developing novel technologies to enhance procedural outcomes^[44,45]^.

## Clinical practice guidelines

The American College of Cardiology and the European Society of Cardiology both provide detailed recommendations for using RFA in AF, one of the most common indications for the procedure. The ACC/AHA/HRS guidelines categorize AF into paroxysmal, persistent, and long-standing persistent subtypes, with RFA being recommended for patients with symptomatic paroxysmal or persistent AF who have failed antiarrhythmic drug therapy^[1]^. The ESC’s guidelines also offer similar indications, endorsing RFA for symptom control in patients who do not respond to pharmacological treatment or prefer procedural intervention^[2]^. Both organizations emphasize that RFA should be considered after weighing the potential benefits against procedural risks, especially in patients with advanced age or comorbidities. The guidelines underscore that patient-specific factors such as age, comorbidities, and arrhythmia burden significantly influence procedural success and complication risk and should be considered during pre-procedural assessment.

For VT, a life-threatening arrhythmia often associated with structural heart disease, the guidelines recommend RFA in cases of drug-refractory symptomatic VT or frequent ICD shocks. The ACC and ESC recognize that RFA can provide substantial arrhythmia control and improve the quality of life for patients with VT, especially in individuals who cannot tolerate antiarrhythmic drugs or experience recurrent VT despite medication^[3]^. The guidelines recommend using electroanatomical mapping and advanced imaging techniques, which enhance the visualization of scar tissue and improve precision in lesion creation. Given the complex anatomy and potential complications associated with VT ablation, they also highlight the need for specialized operator skills and experience. The guidelines recommend high-volume centers with experienced operators for AF and VT to maximize procedural success rates and minimize complications.

Emerging technologies in catheter design, real-time imaging integration, and alternative energy sources have the potential to shape future guideline updates. Contact-force sensing catheters, which provide feedback on the force applied to the cardiac tissue during ablation, have gained prominence recently. The guidelines from both the ACC and ESC recognize the benefit of these devices in optimizing lesion formation and reducing the risk of complications associated with inadequate tissue contact. Contact-force technology has been associated with improved procedural outcomes and a reduced incidence of arrhythmia recurrence, making it likely that future guideline updates may incorporate specific recommendations for its use, especially in complex arrhythmias such as persistent AF and VT^[4]^.

Another area of potential guideline evolution is the integration of real-time imaging modalities such as ICE and MRI. Current guidelines acknowledge the importance of advanced mapping systems for complex cases but do not yet mandate real-time imaging as a standard of care. However, recent studies suggest that ICE and MRI enhance procedural safety and efficacy by providing real-time visualization of cardiac structures and lesion formation, which could reduce complications such as esophageal injury during AF ablation^[5,6]^. As imaging technologies continue to improve, future guidelines may advocate for the routine use of real-time imaging in RFA, particularly for high-risk patients or complex procedures.

The guidelines also discuss adjunctive therapies in conjunction with RFA. Both the ACC and ESC endorse the combination of antiarrhythmic drugs with RFA for AF patients, particularly those with a high risk of recurrence. Emerging research suggests that combining RFA with novel therapies, such as anti-inflammatory agents and hybrid surgical catheter ablation techniques, may improve outcomes in challenging cases^[7]^. Hybrid approaches that integrate catheter-based and surgical ablation are increasingly used for complex cases of AF and VT, particularly in patients with significant atrial remodeling or extensive scar tissue. These techniques offer the advantage of more extensive lesion formation, which could reduce recurrence rates. As hybrid strategies become more widely adopted and supported by clinical evidence, future guidelines will likely incorporate specific recommendations for these therapies, especially for patients with refractory arrhythmias.

Patient selection criteria are integral to the current guidelines, as RFA outcomes are closely linked to factors such as arrhythmia subtype, duration, and structural heart disease. For example, the ACC and ESC guidelines recommend that AF patients with heart failure may derive particular benefit from RFA, as studies have shown improvement in left ventricular function and quality of life post-ablation^[8]^. However, these benefits may not extend to all heart failure patients, as outcomes vary depending on the type and stage of heart failure. Future guidelines may refine patient selection criteria by incorporating predictive tools and scoring systems that comprehensively evaluate individual risk factors and comorbidities. ML algorithms, which analyze large datasets to identify predictors of RFA success, may also shape patient selection guidelines.

Current guidelines stress the importance of procedural safety in managing complications, with recommendations for anticoagulation management, esophageal monitoring, and post-procedural follow-up. The guidelines recommend anticoagulation therapy for AF patients undergoing RFA to minimize thromboembolic risk, with specific protocols for patients with different bleeding risks^[9]^. They also highlight the importance of esophageal temperature monitoring to prevent esophageal injury, a potentially severe complication of AF ablation. Advances in lesion delivery methods, such as cryoablation and PFA, may lead to changes in these guidelines, as early studies suggest these techniques may carry a lower risk of collateral damage compared to traditional RFA. Future guidelines could reflect these findings by recommending alternative energy sources for specific patient populations or arrhythmia types, particularly for cases where traditional RFA poses high risks.

Long-term follow-up is a crucial aspect of RFA care, as arrhythmia recurrence and late complications are not uncommon. Current guidelines recommend regular follow-up visits and Holter monitoring to detect recurrence and assess the need for repeat ablation or medication adjustments^[10]^. With advancements in wearable technology and remote monitoring, future guidelines may advocate for more frequent and comprehensive monitoring options, allowing for continuous rhythm assessment and prompt intervention in cases of recurrence. Remote monitoring tools enable more proactive management of complications and improve patient outcomes by detecting arrhythmia recurrence earlier than traditional follow-up methods.

The influence of emerging technologies will likely prompt additional guideline updates, particularly in data standardization and clinical research. Variability in study design, patient populations, and outcome measures currently complicates the interpretation of RFA research, making it challenging to derive consistent guidance. The guidelines acknowledge these limitations and emphasize the need for standardized definitions of procedural success, complications, and long-term outcomes. Future guidelines may include specific clinical trial design and data reporting criteria, as consistency in outcome measures will be essential for comparing different technologies and establishing best practices^[11]^. By standardizing data collection and reporting protocols, cardiology societies can ensure that guidelines are based on the most reliable evidence and can evolve with technological progress.

## Challenges and limitations

Technical and procedural challenges in RFA involve the complexities associated with the procedure and the technology used. One significant technical challenge is the creation of adequate lesions to isolate or ablate arrhythmogenic foci effectively. The success of RFA heavily relies on the precise application of energy to the targeted tissue, and achieving the appropriate lesion size and depth can be difficult^[1,2]^. Inadequate lesion formation may lead to incomplete ablation, resulting in arrhythmia recurrence or the need for repeat procedures^[3,4]^. The variability in lesion formation is influenced by several factors, including catheter contact force, power delivery, and tissue characteristics^[5,6]^. Innovations such as contact-force sensing catheters have been developed to address these issues by providing real-time feedback on the catheter-tissue interface, thus helping to optimize lesion creation and improve procedural outcomes^[7,8]^. However, these technologies are only sometimes available, and their effectiveness can vary depending on the operator’s experience and the specific clinical scenario^[9,10]^. Another technical challenge is the management of complex arrhythmias, such as VT or AF, which often involve multiple arrhythmogenic foci or reentrant circuits. Precisely mapping and targeting the abnormal electrical pathways are crucial for successful ablation^[11,12]^. Advanced mapping technologies, including electroanatomical mapping and 3D imaging, have significantly improved the ability to visualize and target arrhythmogenic areas. However, these technologies are complex and require specialized training and experience to use effectively^[13,14]^. Integrating these technologies into routine clinical practice remains challenging, particularly in settings with limited resources or expertise. Patient-related factors and complications also pose significant challenges in RFA. One primary concern is the risk of procedural complications, ranging from minor issues to severe adverse events. Common complications include cardiac perforation, which can lead to tamponade and other serious consequences^[15,16]^. Pulmonary vein stenosis is another potential complication, particularly in AF ablation procedures, where damage to the pulmonary veins can impair their function and lead to long-term issues^[17,18]^. Although relatively rare, the risk of esophageal injury is also a concern, particularly in procedures involving lesions near the esophagus^[19,20]^. Strategies to mitigate these risks include improved imaging techniques, careful lesion placement, and balloon-based ablation technologies that may reduce the incidence of complications^[21,22]^. Patient-related factors, such as underlying comorbidities and anatomical variations, can also impact the success and safety of RFA. Patients with structural heart disease, such as cardiomyopathy or previous myocardial infarction, may have altered cardiac anatomy and electrical conduction pathways that complicate the ablation procedure^[23,24]^.

Additionally, patients with frailty or other health issues may have a higher risk of procedural complications and may not tolerate the procedure as well as healthier individuals^[25,26]^. Pre-procedural assessment and risk stratification are crucial to identify patients who may benefit most from RFA and to develop tailored treatment plans that address individual needs and risks^[27,28]^. Variability in outcomes and research findings is another significant challenge in the field of RFA. The success rates of RFA can vary widely depending on the specific arrhythmia being treated, the technology used, and the experience of the operator^[29,30]^. For example, the success rates for AF ablation can range from 60% to 80%, depending on factors such as AF duration, underlying structural heart disease, and advanced mapping technologies^[31,32]^.

Similarly, the success rates for VT ablation can vary depending on the presence of scar tissue, the complexity of the arrhythmia, and the precision of the mapping and ablation techniques^[9,33]^. Research findings on RFA can also be affected by variability in study design, patient populations, and outcome measures. For instance, different studies may use varying definitions of success, such as freedom from arrhythmia or symptom improvement, making it challenging to compare results across studies^[34,35]^. Additionally, the quality and rigor of research can differ, with some studies needing more robust methodologies or long-term follow-up, which can impact the reliability of their findings^[36,37]^. Efforts to standardize outcome measures and improve the quality of research are essential to enhance the evidence base for RFA and to provide more consistent and reliable guidance for clinical practice^[38,39]^.

## Patient selection and complications management

Selecting the right patients for RFA begins with a thorough understanding of the indications for the procedure. RFA is primarily indicated for patients with symptomatic arrhythmias that are refractory to medical therapy. AF and VT are two of the most common arrhythmias treated with RFA. In the case of AF, the procedure is typically considered in patients who experience persistent symptoms despite adequate pharmacological management or in those who have experienced significant adverse effects from antiarrhythmic medications^[109]^. Similarly, patients with VT, particularly those with structural heart disease or recurrent episodes, may benefit from RFA when they are symptomatic and at risk of more severe outcomes. The ideal candidate for RFA often exhibits a few specific characteristics. For instance, patients with paroxysmal AF – those whose symptoms come and go – tend to have better outcomes following RFA than those with persistent or long-standing persistent AF^[40]^. Studies have shown that success rates for RFA in paroxysmal AF can exceed 80%, while rates for persistent AF may range between 60% and 70%. This difference underscores the importance of careful patient selection based on the type and duration of the arrhythmia. Moreover, underlying structural heart disease must be considered when evaluating candidates for RFA^[110]^. Patients with conditions such as hypertrophic cardiomyopathy, previous myocardial infarction, or significant left ventricular dysfunction may present unique challenges during the procedure. While RFA can still be effective in these populations, a comprehensive assessment of their cardiac status is essential for predicting outcomes and managing potential complications. In addition to structural considerations, patient comorbidities play a significant role in determining candidacy for RFA^[89]^. Factors such as age, obesity, diabetes, hypertension, and pulmonary disease can influence both the risk of procedural complications and the likelihood of arrhythmia recurrence post-ablation. For instance, older patients or those with multiple comorbidities may face higher risks during the procedure and may not tolerate it, as well as healthier individuals. Therefore, a detailed pre-procedural assessment – including a review of medical history, physical examination, and appropriate imaging studies – is crucial for identifying candidates most likely to benefit from RFA^[111]^. Once suitable candidates are identified, it is essential to consider potential complications associated with the RFA procedure. Complications can range from minor to severe, and effective management strategies are necessary to minimize risks. One of the most common complications is the recurrence of arrhythmias, which can occur due to incomplete ablation or multiple arrhythmogenic foci. Incomplete lesions may lead to reentrant circuits that trigger arrhythmia recurrence, highlighting the importance of achieving adequate lesion formation during the procedure. Pre-procedural mapping techniques such as electroanatomical mapping can be invaluable in managing the risk of recurrence^[112]^. These technologies allow for detailed visualization of electrical activity within the heart, helping operators accurately target arrhythmogenic substrates. Additionally, post-procedural follow-up is critical for monitoring patients for signs of recurrence. Clinicians may utilize regular electrocardiograms (ECGs) or Holter monitoring to assess for any arrhythmias that may emerge after the procedure, enabling timely interventions if necessary. Another significant complication associated with RFA is thromboembolism, particularly in patients undergoing AF ablation. The risk of thromboembolic events arises from the potential for thrombus formation in the left atrial appendage (LAA) during the procedure^[9]^. Patients with a history of thromboembolic events or those who have significant risk factors – such as advanced age, hypertension, diabetes, or heart failure – may require anticoagulation therapy both before and after the procedure to mitigate this risk. Studies have shown that utilizing anticoagulation can significantly reduce the incidence of stroke and other thromboembolic complications associated with RFA. Management strategies for thromboembolism involve a multidisciplinary approach, where cardiologists, electrophysiologists, and primary care physicians collaborate to optimize anticoagulation protocols. Guidelines recommend careful assessment of the patient’s bleeding risk versus the thromboembolic risk when determining the appropriate anticoagulation regimen^[113]^. For instance, patients with a high risk of thromboembolism may be placed on direct oral anticoagulants (DOACs) or vitamin K antagonists, carefully monitoring renal function and coagulation parameters to ensure therapeutic efficacy while minimizing bleeding risks. Effective patient education is also a vital aspect of managing complications. Providing patients with detailed information about the signs and symptoms of potential complications can empower them to seek timely medical attention. For example, patients should be educated on the signs of stroke, such as sudden weakness, speech difficulties, or altered mental status, and advised to contact emergency services immediately if these symptoms arise^[114]^. Additionally, patients should be informed about the importance of adhering to their prescribed anticoagulation therapy and attending follow-up appointments for regular monitoring. Illustrative case studies can provide valuable insights into the practical management of patient selection and complications in RFA. In one case, a 65-year-old male with a history of paroxysmal AF and hypertension was referred for RFA after experiencing recurrent symptoms despite maximal medical therapy. Pre-procedural assessment revealed normal left ventricular function and no significant structural heart disease. The procedure was successfully performed using electroanatomical mapping, isolating the pulmonary veins^[115]^. Post-procedure, the patient was placed on anticoagulation therapy for 30 days and monitored closely for any arrhythmia recurrence. Follow-up at 6 months showed no arrhythmia recurrence, highlighting the importance of careful patient selection and effective management strategies in achieving favorable outcomes. In another case, a 70-year-old female with persistent AF and a history of transient ischemic attacks (TIAs) was considered for RFA. Given her increased risk of thromboembolism, a comprehensive pre-procedural evaluation was conducted, including a transesophageal echocardiogram to assess for LAA thrombus^[116]^. The absence of a thrombus allowed the procedure to proceed safely. Anticoagulation was initiated before the procedure and continued for three months post-ablation. Despite initial procedural success, the patient experienced recurrent AF after three months, necessitating additional mapping and ablation. This case underscores the challenges of managing recurrent arrhythmias, particularly in patients with significant risk factors. The variability in patient outcomes following RFA reflects the complexity of arrhythmia management^[117]^. The selection process, management of complications, and overall approach to care must be tailored to each patient’s unique circumstances. Ongoing research is essential to enhance our understanding of factors influencing procedural success and further refine patient selection criteria. This includes investigating novel adjunctive therapies that may improve outcomes, such as integrating pharmacological agents or other interventional procedures alongside RFA^[118]^.

## Future directions

Emerging RFA technologies are rapidly advancing, with innovations in catheter design, mapping systems, energy delivery methods, and AI significantly enhancing procedural outcomes. One notable innovation is the development of contact-force sensing catheters, which provide real-time feedback on the force exerted by the catheter tip on cardiac tissue^[100]^. This technology helps optimize lesion creation by ensuring adequate contact force and improving the precision and consistency of ablation. Contact-force sensing has been shown to reduce complications and enhance procedural outcomes, contributing to more effective and safer RFA. Moreover, integrating advanced 3D mapping systems allows for detailed visualization of cardiac electrical activity and precise localization of arrhythmogenic substrates^[101]^. High-density mapping techniques have improved the accuracy of ablation procedures, leading to better outcomes for complex arrhythmias such as AF and VT. The development of alternative energy sources, such as cryoablation, offers a significant advancement. Cryoablation uses extreme cold to create lesions, which has shown promise in reducing complications associated with traditional radiofrequency energy, such as pulmonary vein stenosis, potentially providing a safer option for certain patients^[102]^. Laser ablation and high-intensity focused ultrasound (HIFU) are also being explored as alternative methods for creating lesions, enhancing precision, and reducing procedural risks. The continued evaluation of these technologies will be crucial in determining their roles in clinical practice and their impact on patient outcomes. AI-driven algorithms transform RFA through more precise ablation path planning and real-time procedural analysis, enhancing accuracy and safety^[103]^. AI plays a vital role in pre-procedural mapping, where algorithms analyze patient-specific imaging data to suggest optimal catheter trajectories and target arrhythmogenic zones accurately. This individualized approach enables clinicians to create effective lesion patterns, reducing the likelihood of arrhythmia recurrence and enhancing long-term outcomes. Furthermore, AI-based algorithms dynamically adjust parameters such as energy output and catheter position during the procedure, facilitating more consistent lesion formation and reducing procedural time. AI also holds potential in post-procedural analysis, providing insights into lesion quality and identifying areas where ablation may have been suboptimal, ultimately enhancing long-term efficacy^[104]^. Advanced imaging modalities, including real-time ICE and MRI, revolutionize RFA by offering high-resolution, detailed visualization of cardiac structures and lesion formation. MRI provides unique insights into tissue characteristics that can improve patient-specific ablation planning and enable more precise targeting of arrhythmogenic substrates. ICE enhances visualization of catheter-tissue contact, ensuring adequate pressure and alignment during ablation, which is crucial for creating effective lesions without unnecessary tissue damage. Combined with AI, these imaging modalities enhance the accuracy and efficiency of RFA procedures. AI-driven image segmentation allows for more accurate mapping of arrhythmogenic regions, minimizing the risk of incomplete lesions^[57]^. Additionally, AI-enhanced imaging analysis helps clinicians monitor lesion progress during ablation, ensuring procedural effectiveness while minimizing complications. For example, advanced fluoroscopy with AI-based motion compensation provides real-time adjustments, reducing radiation exposure and improving catheter guidance. These advancements in AI and imaging are poised to drive significant improvements in RFA outcomes, making ablation more personalized, efficient, and safe for patients. Areas requiring further research include optimizing patient selection criteria and developing personalized treatment approaches^[105]^. Current research focuses on identifying predictors of success and tailoring RFA procedures to individual patient characteristics, such as AF duration and underlying structural heart disease. Refining patient selection and developing personalized treatment strategies can improve the likelihood of successful outcomes and reduce complications. Additionally, exploring novel adjunctive therapies to enhance RFA effectiveness is essential. For instance, combining RFA with pharmacological agents or other interventional procedures may improve outcomes for patients with persistent or recurrent arrhythmias^[106]^. Research into genetic and molecular markers may also play a role in predicting RFA success and guiding treatment decisions, allowing for more tailored approaches and optimized outcomes. Integrating real-time imaging technologies may provide additional insights into lesion formation, facilitating more accurate targeting of arrhythmogenic areas and enhancing overall procedural safety. Ongoing research will be critical in identifying and mitigating risks associated with RFA, such as cardiac perforation and esophageal injury. Advances in imaging and monitoring technologies will continue to play a vital role in improving procedural safety and reducing the incidence of adverse events, ultimately contributing to the future of RFA and enhancing patient care^[64]^.

## Long-term efficacy

One significant trial is the CABANA trial (Catheter Ablation vs. Anticoagulation for Atrial Fibrillation), which aimed to determine whether RFA improves outcomes compared to conventional therapy in patients with AF. The trial enrolled over 2,000 patients and found that RFA was associated with a lower incidence of death and stroke compared to standard medical therapy, particularly in patients with symptomatic AF^[95]^. However, the study also highlighted that while RFA effectively reduces symptoms and improves quality of life, recurrence rates of AF can still be significant, necessitating careful long-term follow-up and potential retreatment in some cases. Long-term data from the PRECEPT study (Predicting Recurrence of Atrial Fibrillation after Catheter Ablation) indicate that approximately 50% of patients experience arrhythmia recurrence within five years post-RFA^[96]^. This highlights the importance of patient selection, as those with persistent AF or structural heart disease tend to have higher recurrence rates. Identifying predictors of recurrence, such as AF duration, age, and left atrial size, is crucial for optimizing patient outcomes. Hybrid ablation techniques, which combine RFA with surgical approaches, have emerged as a promising strategy for managing complex cases of AF^[97]^. Studies have shown that hybrid approaches, such as the combination of minimally invasive surgical ablation with percutaneous RFA, can yield superior long-term results, particularly in patients with persistent or long-standing AF. For instance, the HRS/ACC/AHA guidelines recommend hybrid ablation for patients who have failed prior RFA or who have complex arrhythmias that are challenging to manage with RFA alone. Combining RFA with drug therapies has also shown potential for improving long-term outcomes^[98]^. Antiarrhythmic medications with RFA can help maintain sinus rhythm and reduce recurrence rates. A recent study found that patients receiving a combination of RFA and antiarrhythmic drugs had significantly lower arrhythmia recurrence rates than those who underwent RFA alone. Furthermore, ongoing research into the use of novel pharmacological agents, such as potassium channel blockers and agents targeting fibrosis, may enhance the effectiveness of RFA and improve long-term outcomes^[99]^.

## Concluding remarks

The field of RFA in cardiology has experienced substantial progress over the past few decades, significantly enhancing the management of various cardiac arrhythmias. This literature review highlights the clinical efficacy, technical advancements, and ongoing challenges associated with RFA. RFA has become a cornerstone in treating arrhythmias such as AF, atrial flutter, and VT, with success rates that continue to improve thanks to innovations in catheter technology, mapping systems, and energy delivery methods. Despite these advancements, several challenges remain. Technical and procedural difficulties, patient-related factors, and variability in outcomes pose significant barriers to the consistent success of RFA. These challenges underscore the need for ongoing research and development to optimize procedural techniques, improve patient selection, and reduce complications. The future of RFA is promising, with several key innovations on the horizon. Advancements in catheter design are set to revolutionize the field by developing next-generation catheters that feature improved flexibility, steerability, and enhanced energy delivery capabilities. These advancements will allow for more precise targeting of arrhythmogenic foci and improved lesion formation, which is critical for successful ablation outcomes. Integration with real-time imaging technologies, such as MRI and ultrasound, will further enhance the safety and effectiveness of RFA procedures. These imaging modalities can provide clinicians with detailed anatomical information, allowing for better visualization of the heart’s structures and facilitating accurate lesion placement. Such integration will help in the early identification of complications and ensure that patients receive tailored treatments based on their unique anatomical and electrophysiological profiles.

Moreover, innovative lesion delivery methods, including cryoablation and alternative energy sources, are gaining traction. These techniques can minimize collateral damage to surrounding tissues and improve patient safety, particularly in high-risk populations. The exploration of hybrid approaches, which combine RFA with surgical techniques, is also paving the way for enhanced treatment strategies, particularly for patients with complex arrhythmias.

## Call to action

To further advance the RFA field in cardiology, the medical and scientific communities must continue prioritizing research and innovation. Clinicians and researchers should focus on developing and refining emerging technologies, such as advanced mapping systems and alternative energy sources, to enhance procedural outcomes and reduce complications. Additionally, greater emphasis should be placed on personalized medicine approaches, ensuring that RFA treatments are tailored to individual patient profiles for optimal effectiveness. Collaboration across multidisciplinary teams, including cardiologists, biomedical engineers, and researchers, drives these innovations. Moreover, large-scale clinical trials and studies should be conducted to validate new techniques and technologies, providing robust evidence to guide clinical practice. Healthcare institutions and policymakers must also support these efforts by investing in research, training, and disseminating best practices. Ultimately, the goal is to ensure that RFA continues to evolve as a safe, effective, and accessible treatment option for patients worldwide, significantly improving the management of cardiac arrhythmias and enhancing patient outcomes.

## Data Availability

This published article and its supplementary information files include all data generated or analyzed during this study.
